# Interindividual differences in auditory processing moderate the effect of auditory-motor coupling on paired-associate learning

**DOI:** 10.1038/s41598-025-23360-w

**Published:** 2025-10-17

**Authors:** Maren Schmidt-Kassow, Johannes Kasper, Camilla Diefenbach, Jochen Kaiser

**Affiliations:** 1 Department of Psychiatry, Psychosomatic Medicine and Psychotherapy, Goethe University Frankfurt, University Hospital, Heinrich-Hoffmann-Str. 10, 60528 Frankfurt, Germany; 2 Faculty of Medicine, Institute of Medical Psychology, Goethe University, Frankfurt, Germany; 3 Cooperative Brain Imaging Center, Goethe University, Frankfurt, Germany

**Keywords:** Language, Consolidation, Long-term memory

## Abstract

The present study explored the effects of auditory–motor coupling and individual differences in pitch perception on verbal learning. Participants (N = 47) learned a list of foreign-language vocabulary word pairs under isochronous or self-initiated auditory stimulation while cycling on a stationary bike. As expected, better movement consistency—i.e., lower cycling variability and more stable synchronization with the vocabulary presentation—was weakly associated with improved memory performance. Interestingly, we found inter-individual differences in the pattern of results: participants with fundamental pitch perception (FPP) benefited from isochronous stimulation immediately after learning (Day 1), but not after overnight consolidation (Day 2), whereas participants with spectral pitch perception (SPP) showed a long-term memory benefit from isochronous stimulation. This suggests that auditory processing characteristics modulate whether isochronous input supports early encoding or later consolidation. Furthermore, musically experienced participants showed more variable pedaling in the self-initiated condition, possibly reflecting deliberate modulation of input timing. In contrast, isochronous stimulation reduced variability across all participants, indicating implicit synchronization even without an explicit instruction to synchronize. Overall, these findings highlight the role of individual auditory and motor traits in shaping the cognitive benefits of sensorimotor coupling during learning.

**Supplementary Information:**

The online version contains supplementary material available at 10.1038/s41598-025-23360-w.

## Introduction

Over the past two decades, numerous studies have examined the potential effects of acute physical activity on memory. These studies have explored the impact of exercise before, during, and after the encoding of new material, as well as the role of exercise intensity in this process. While some findings have suggested that physical activity during encoding may not significantly influence later retrieval, others have produced mixed results^1,2^. Earlier studies from our laboratory have observed a positive effect of simultaneous movement on auditory paired-associate learning, i.e., learning of new foreign-language vocabulary^3–5^. This was true for both moderate^3,5^ and very low-intensity movement^4^, and especially for people with a poorer learning performance^5^.

Based on these findings, we conducted a series of electroencephalography (EEG) studies^6–9^ to investigate the neural underpinnings of auditory-motor coupling on stimulus encoding. Our EEG studies have demonstrated repeatedly that isochronous stimulation led to a stronger P300 response in an auditory oddball paradigm and that the precision of auditory-motor synchronization (AMS) correlated positively with P300 amplitude. Since larger P300 amplitudes have been associated with superior information processing^10^, we believe that AMS may result in more efficient stimulus encoding compared to a physically passive condition. However, self-initiated stimulation, i.e., a sound presentation naturally aligned with movement execution without deliberate synchronization effort, did not lead to a stronger P300 response^6^. Up until now we lack behavioral data on the effect of self-initiated stimulation on long-term memory in comparison to AMS. Therefore, the present study explored whether the effect of AMS on P300 amplitude found in EEG is reflected in memorization differences of new vocabulary in a paired-associate learning paradigm. We opted for a within-subject design to compare the memorization of new vocabulary encoded under two distinct sensory-motor contingency conditions: a putative AMS condition in which subjects were informed that they could synchronize their pedaling movements with isochronous auditory stimuli, and a self-initiated stimulation condition in which stimulus presentation was triggered by the subjects’ own pedaling movements. To tap into the dynamics of long-term memory formation, we measured vocabulary recall twice, on the same day after encoding (Day 1) and 24 h later (Day 2).

AMS is based on at least two mechanisms: First, it requires the accurate identification of the underlying rhythm in a stimulus sequence (auditory component). Second, it relies on the ability to align motor execution with the auditory rhythm (motor component). We assessed the latter through continuous recording of the synchronization performance during the experiment. To gain insights into the first component, we utilized an auditory test that has been shown to correlate with rhythmic auditory processing preferences. Previous research has shown that people vary in the way they perceive multi-harmonic sounds with “missing fundamentals” (MF), i.e., without spectral energy at the frequency of the periodicity (*F*_*0*_)^11^. The pitch perception preference is tested by presenting pairs of complex sounds whose fundamental tone frequency has been removed. This paradigm results in an individual pitch perception index ranging from -1 to 1. A pitch index of -1 indicates that a participant perceives the pitch of the complex sounds always at the implied (missing) fundamental frequency (periodicity pitch), called “fundamental pitch perceiver” (FPP), whereas a pitch index of + 1 characterizes people who always rely on the spectral envelope of the sound to determine its pitch class, called “spectral pitch perceiver” (SPP). Most subjects display a mix of both perceptual strategies, depending on the number, order and frequency of the harmonics (with a pitch index of 0 corresponding to a 50/50 odds ratio). Interestingly, pitch perception preferences have been found to be associated with the preferred musical style and in musicians with the type of musical instruments played^12^. FPPs seemed to prefer instruments producing percussive or high-pitched tones, characterized by brief, sharp, or impulsive sounds (e.g., drums, guitar, piano, trumpet, or flute) and high rhythmicity. In contrast, SPPs typically played musical instruments of a lower pitch that produce sustained sounds with characteristic changes in timbre (e.g., bassoon, saxophone, French horn, violoncello, or organ).

Pitch perception preference has also been linked to anatomical differences of Heschl’s gyrus^11^. Heschl’s gyrus plays a pivotal role in pitch perception, with different parts of this region specialized for processing fundamental and harmonic pitch information. SPPs showed larger gray matter volume and increased fMRI activity in lateral Heschl’s gyrus of their *right* compared with their left hemisphere, whereas the opposite pattern was found in FPPs^12^. These findings are consistent with the hypothesis that the left hemisphere is specialized for processing rapid temporal information, whereas the right hemisphere is implicated in spectral processing^13,14^.

Importantly for the present study, pitch perception preference has been shown to be independent of formal musical training^12,15,16^ (for details see supplemental information, suppl. Table 2). This suggests that it reflects a stable, possibly innate, auditory processing predisposition rather than a learned skill. As such, the MF paradigm provides a valuable tool for probing basic auditory mechanisms that are not significantly shaped by experience.

Thus, the pitch perception test may tap into auditory predispositions linked to rhythm perception or processing preferences, as well as neuroanatomical variations. Namely, FPPs may be more sensitive to rhythmic precision in comparison to SPPs. We therefore hypothesized that FPPs may also demonstrate superior synchronization abilities and less temporal variability in a self-initiated stimulation condition. Concurrently, they may show a smaller difference in memory performance between the AMS and the self-initiated condition compared to SPPs.

On the other hand, we anticipated that SPPs could derive greater benefit from the active synchronization with an external rhythmic structure in the AMS condition, given their perceptual preference for (slowly varying) spectral aspects of sounds and consequently a reduced focus on auditory rhythmicity. Evidence from patients with basal ganglia lesions^17^ suggests that an external rhythm can enhance other cognitive functions despite impaired rhythm perception. Based on these observations, we expected inter-individual performance differences in the auditory paired associative learning task based on pitch perception preferences. Specifically, SPPs, i.e., healthy individuals with a perceptual preference for spectral rather than temporal sound characteristics, should show a positive effect of an external rhythmic structure on long-term memory.

### Hypotheses

1. Similar to our EEG results on P300 amplitude, we expected a main effect of stimulation condition on memory recall, i.e., better performance with isochronous stimulation (resulting in AMS) compared to self-initiated stimulation.
2. The beneficial effect of memory performance under isochronous compared to self-initiated stimulation should correlate positively with the pitch perception index, i.e., the more the spectral pitch perception mode dominates, the more benefit should be gained from active AMS. This benefit may primarily materialize in long-term memory formation, i.e., in less forgetting over night with isochronous compared to self-initiated stimulation in SPPs (2-way interaction between stimulation condition and pitch index, or 3-way interaction between stimulation condition, testing day and pitch index).
3. The learning outcome in the isochronous condition should correlate positively with synchronization performance independently of pitch perception preference (interaction between stimulation condition and movement synchronization on memory recall).
4. FPPs should show a better synchronization performance than SPPs in the isochronous stimulation condition (main effect of pitch perception preference on movement synchronization, defined only in the isochronous stimulation condition) and a lower temporal variability in the self-initiated condition (interaction between stimulation condition and pitch perception preference on pedaling variability).

## Materials and methods

### Participants

A total of 48 participants between the ages of 18 and 30 (Mean: 23.6; *SD*: 2.8) were included in the study. To avoid gender effects, the same number was recruited for both genders (mean age men: 22.9; *SD*: 2.25; mean age women: 24.3; *SD*: 3.2).

Participants were students or trainees and self-reported right-handers. Apart from hormonal contraception, participants did not take any regular medication, were healthy and of normal weight according to the WHO definition with an average BMI (body mass index: kg/m^2^) of 22.5 (*SD*: 2.3) (M women: 21.8; *SD*: 2.7; M men: 23.2; *SD*: 1.6). All participants were native German speakers, grew up monolingually and spoke up to five foreign languages (M: 2.5, median 2.0; *SD*: 1.03). Because the experiment involved learning Polish words, candidates with Polish or Slavic language skills and a stay of more than two weeks in a Slavic-speaking country were excluded. The acquisition of the stated foreign languages had all begun after the age of 6. They played up to four musical instruments (M: 1.4, median 1.0; *SD*: 1.14).

One participant was excluded from statistical analyses due to a very poor performance in the vocabulary tests (only 1 correct answer in the first test per session). This resulted in a data set of 47 participants.

### Ethics statement

The ethics committee of the Faculty of Medicine, Goethe University Frankfurt am Main, approved the research project “The influence of synchronous physical activity on brain plasticity and foreign language learning”, which included the present study. All procedures were conducted in accordance with the Declaration of Helsinki. All participants were informed orally and in writing about the aims of the project and provided their written consent. Informed consent was obtained from all subjects. Withdrawal of study participation was possible at any time and without giving reasons. A remuneration of €10 per hour was paid after all parts of the study had been completed.

### Experimental design and procedure

We used a 2*2 within-subjects design with the experimental factors *stimulation condition* (isochronous stimulation vs. self-initiated stimulation) and *testing day* (immediately after encoding—day 1 vs. 24 h later—day 2). The main between-subjects factor of interest was the pitch perception index measured during a pre-experimental screening session. The main outcome variables of interest were vocabulary recall rate and pedaling timing.

Participants came to our laboratory on three days: following the screening session (see below), two learning sessions of 45–50 min each were held exactly 72 h apart. The measurements were taken between 12 noon and 8 pm in a low-stimulus environment.

In each learning session, participants were asked to learn 40 unique Polish-German vocabulary pairs (80 in total) while cycling on an ergometer. The vocabulary pairs were either played at a fixed rate (isochronous stimulation) or self-initiated by the pedaling. Participants were then tested for vocabulary recall and again 24 h later at home. At the end of both learning sessions, participants completed a short 5-item questionnaire asking about the subjective effort of the task and the ease of keeping the cycling speed, the previous night’s sleep duration and quality, as well as about general stress. The order of the stimulation conditions was pseudo-randomized and balanced across participants while making sure that pitch index was roughly homogeneously distributed across condition orders (t_45_ of the difference between orders ≈ 0.125, p ≈ 0.9, see suppl. Figure 1).

### Pre-experimental session

In the pre-experimental session, participants completed a questionnaire asking for their age, gender, number of foreign languages spoken and instruments played (including the duration) and a questionnaire on physical fitness. Cardiovascular diseases and restrictive injuries to the bone, muscle or ligament apparatus were ruled out. Additionally, participants were tested for their auditory learning ability. To this end, they listened to 40 German pseudowords and were asked to perform a written test afterwards. Pseudo-vocabulary refers to words that could exist in the German language in terms of their phonetic composition, but do not actually exist. Participants who remembered more than 20 out of a total of 40 words correctly were excluded from the study to prevent a ceiling effect.

To determine the individual dominant mode of auditory pitch perception^16^ participants then performed a psychophysical test^11^, which took about 22 min.

All data were anonymized, and each participant was given a consecutive subject number. Participants were asked to maintain a consistent lifestyle in terms of their diet, exercise and sleep in the week before the two learning sessions and not to drink alcohol the evening before the learning sessions.

#### Pitch perception preference

Individual pitch perception was characterized by measuring the relative prevalence of fundamental pitch (*F*_*0*_) perception of acoustically ambiguous multi-harmonic “missing fundamental” (MF) sounds. MF sounds lack energy at the lower harmonics, i.e. at the root frequency *F*_*0*_ (harmonic order *n* = 1), and a varying number of higher harmonics up to *n*_*min*_, the lowest harmonic with spectral energy. In the pitch test, the parameters* n*_*min*_, *F*_*0*_ and the number of harmonics, *N,* were systematically varied^11^. Depending on inter-individually varying properties of the auditory system^11^ and sound parameters like frequency and harmonic order *n*_*min*_^16^, the pitch of such sounds is either perceived at the periodicity frequency (fundamental pitch or *F*_*0*_ perception) or at the frequency of a harmonic contained in the spectrum, often assumed to be *n*_*min*_ (spectral pitch or *F*_*SP*_ perception). In the test, 324 unique sounds were presented once in 162 pairs (duration 0.6 s per sound, inter-stimulus interval 0.4 s, linear fade-in and fade-out of 10 ms), such that the periodicity frequency, *F*_*0*_, changed in the opposite direction of the spectral frequency *F*_*SP*_, while the frequency of the highest harmonic was kept constant within a pair to minimize the perception of “edge pitch”.

The test was implemented in MATLAB R2010a and carried out on a laboratory computer (Fujitsu ESPRIMO P2560, MI4W—D3041). Sounds were synthesized within the routine with a spectral roll of of − 5 dB per harmonic and an additional log-linear attenuation of − 3 dB from 1 to 3 kHz and of − 17 dB from 3 to 15.9 kHz, and presented binaurally over Philips SHP 2000 headphones driven by an ASIO EDIROL FA-66 sound card at an individually set comfortable volume. A GUI presented instructions and the progress, and answers were given with the left (for “first tone higher”) or right (for “second tone higher”) arrow keys on a keyboard. Participants were instructed to indicate their first spontaneous impression. Answering triggered playback of the next sound pair. The order of pairs as well as the order of sounds within a pair (and therefore the relative pitch direction) were pseudo-randomized but kept constant for all participants.

The average pitch perception mode per participant was calculated as δ_P_ = (n(*F*_*SP*_) – n(F_0_*))/*(n(*F*_*SP*_) + n(*F*_*0*_)), the “pitch perception index” according to Schneider et al.^11^ ranging from − 1 (extreme fundamental pitch perception) to + 1 (extreme spectral pitch perception).

### Learning sessions

Participants underwent two vocabulary learning sessions corresponding to two experimental conditions while cycling on an ergometer (Dynavit® Conditronic 100). In each session, the pedaling resistance was initially set at 25 watts and was increased to 40 watts by most participants to ensure a comfortable intensity of exercise.

In the isochronous stimulation condition vocabulary pairs were presented at a fixed rate of 0.5 Hz corresponding to an isochronous stimulus-onset asynchrony (SOA) of 2 s. Participants were cued into a pedaling rate (cadence) of twice that rate by a preceding sequence of sine tones with an initial 1 Hz presentation rate (see below). Participants were informed that they were free to synchronize with the stimuli if they perceived this as helpful. However, to minimize dual-task interferences, no explicit instruction to synchronize was given, as the primary objective of the task was the acquisition of new vocabulary rather than the alignment with an auditory stimulus. Consequently, while the isochronous stimulation condition may result in auditory–motor synchronization (AMS), it may also reflect a condition of simultaneous movement without synchronization, depending on the individual participant. This is consistent with findings by Kern et al.^18^, who showed that individuals differ systematically in their preferred auditory temporal processing regimes and AMS tendencies, indicating that synchronization likelihood and efficacy can vary across participants even under identical rhythmic conditions. Furthermore, prior work by Assaneo et al.^19^ has demonstrated that spontaneous synchronization to speech rhythm correlates with enhanced language learning performance, suggesting that participants who incidentally synchronized with the isochronous stimuli may have benefited from temporally aligned auditory-motor coupling. For the isochronous stimulation condition, one learning session lasted 28 min.

In the self-initiated condition, vocabulary presentation was triggered by the pedaling. Every second time the left pedal crossed the light barrier, a sound (word or sine tone) was played. Accordingly, participants chose a self-determined and potentially varying vocabulary presentation rate. The duration of this learning session varied between participants, with an average duration of 26 (*SD*: 3.3) minutes. Accordingly, cycling durations were closely matched across condition (isochronous: 28 min; self-generated: 26 min).

In each learning session, 40 Polish-German vocabulary pairs were presented to the participants via headphones (Philips SHP 2000) twice, in two blocks per session, while cycling. The Polish and German words had been recorded by a native speaker of the respective language with phonetic-linguistic knowledge. The vocabulary lists for each learning session contained 20 nouns and 20 verbs. Polish was chosen because it is phonetically distinct from German, containing many phonemes not occurring in German, which makes it difficult to learn for German native speakers^20^.

**Fig. 1:**
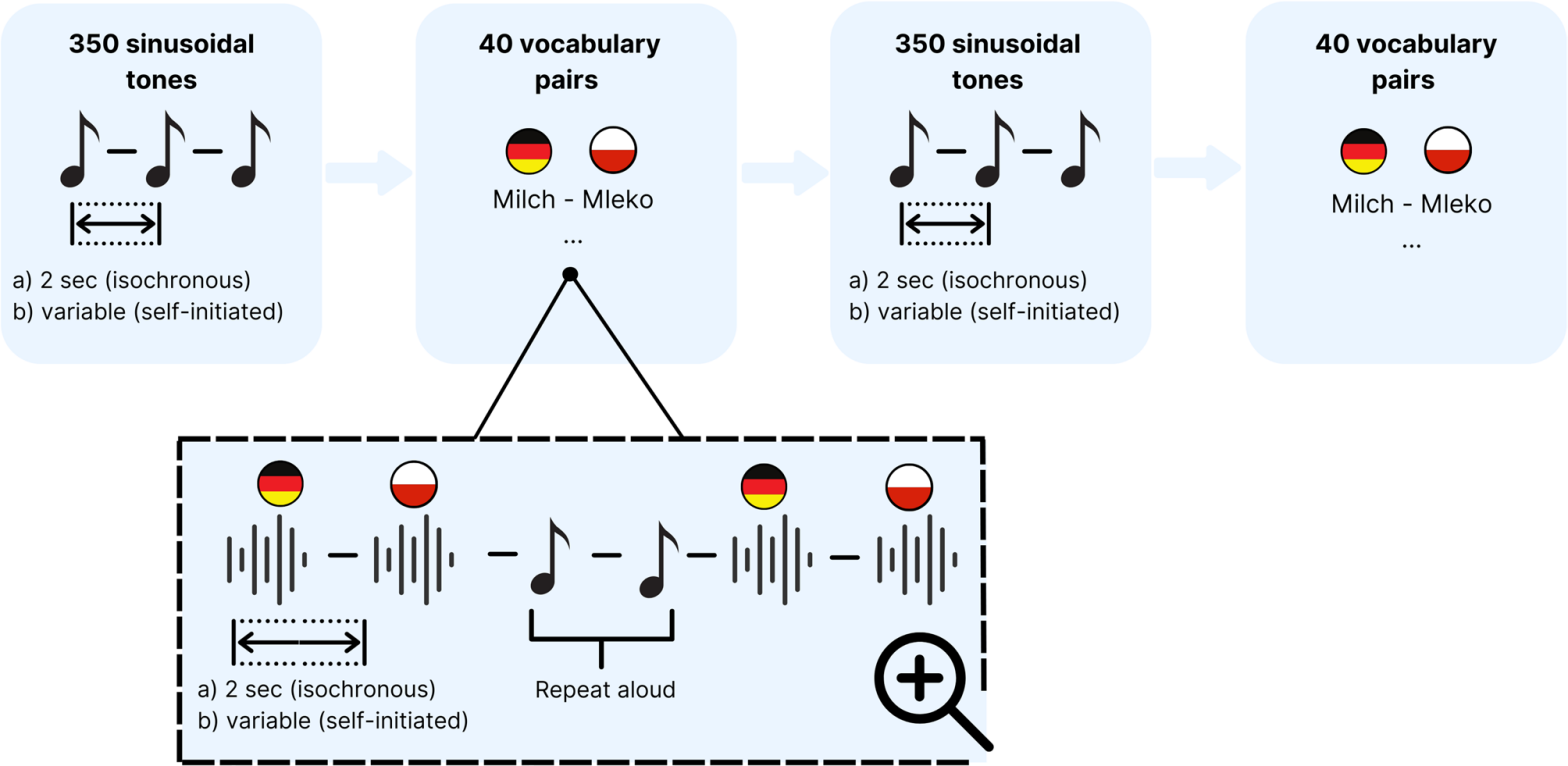
Schematic overview of the encoding phase in the isochronous (**a**) and self-initiated (**b**) stimulation conditions.

To accustom participants with the simultaneous motor activity and auditory input, prior to listening to the vocabulary pairs, participants listened to 350 sinusoidal tones with a frequency of 650 Hz and a duration of 50 ms at a self-initiated or fixed (depending on the respective condition) presentation rate (initially 1 Hz and later 0.5 Hz) while already cycling (see Fig. 1). After 350 tones, three high-frequency sinusoidal tones with a frequency of 700 Hz signaled the start of the first block of vocabulary presentation. Each vocabulary pair was followed by two sinusoidal tones, synchronously to which participants repeated the two last heard words aloud to preclude the use of individual memory aids. The first block was followed by a seven-minute learning break during which participants continued cycling while sinusoidal tones were presented at a self-initiated or fixed (0.5 Hz) presentation rate, depending on the condition. Again, three high-frequency sinusoidal tones announced the start of the second block of vocabulary presentation. This was the same list of vocabulary pairs as in the first block but played in a new randomized order. Therefore, each participant listened to each vocabulary pair twice. The vocabulary lists (with 40 vocabulary pairs each) were balanced across participants between the conditions.

Immediately following the second vocabulary block, participants watched a 10-minute silent film “Shaun, the sheep” to prevent a recency effect 18, a well-established memory recall advantage of the last item in a sequence. After each encoding session, participants completed two auditory vocabulary tests (i.e., a total of four tests), one test immediately following the silent film (using the headphones used during the learning blocks, “Day 1”) and the same test again 24 hours later from home (using individual headphones, “Day 2”). Each of the 40 Polish words were presented once and the participants were asked to type the corresponding German translation, if remembered. The test could only be completed once in a time span of 6 minutes and 40 seconds, with the remaining time continuously displayed on the left-hand side of the screen. Once the allowed timespan had elapsed, the test automatically closed and could not be restarted. The time limit was calibrated to give participants enough time to think, while preventing them from looking up words. Playback volume could be adjusted individually. Participants could not see the result of their test.

On-line access to the second vocabulary test was only activated exactly 24 h later within a time window of 2 h. Again, only one attempt was possible and the results could not be accessed.

### Data collection

To assess motor performance, we measured the cycling cadence by recording the voltage of the photoelectric cells of the light barrier with a sampling rate of 100 Hz and detecting the time points when the left pedal crossed the light barrier. From the resulting vector of timestamps, we derived two summary metrics per subject and session: the pedaling speed in Hz as the average inverse revolution duration, and the coefficient of variation (CV) as the standard deviation of the instantaneous speed (i.e., the inverse revolution duration) divided by the mean speed to quantify the variability in motor execution. In addition, to measure synchronization performance to the isochronous stimulation, we calculated the inter-beat deviation (IBD) as the mean absolute deviation between the revolution duration and half the SOA (which was always 1 s). Under the self-initiated stimulation, this value was zero.

To assess the learning performance, we counted the number of recalled words as the number of correctly entered German translations in each of the four vocabulary tests, where the theoretical maximum* n*, i.e., the number of trials in each test, was 40.

### Statistical analyses

#### Memory performance

We modeled the observed number of successfully recalled items in each of the four vocabulary tests per subject as a binomially distributed random variable with the recall probability rate *p*_*i*_ conditional on the stimulation condition (self-initiated vs. isochronous, *hypothesis 1*), the testing day (Day 1, immediately after encoding vs. Day 2, 24 h later) and the pitch index (*hypothesis 2*), and various control covariates within a generalized multilevel Bayesian regression framework using the R package *brms*^21^. In particular, the expected value of the log-odds (logit) of the recall rate was modeled as a linear combination of an intercept drawn from a Gaussian distribution over subjects whose standard deviation σ is estimated from the data (“random intercept”), binary effects for stimulation condition and testing day also drawn from a multivariate Gaussian distributions over subjects (“random slopes”) as well as a population-level (“fixed”) effect for the interaction between these two factors. In addition, a population-level non-linear smooth effect (penalized thin plate splines) of pitch index as well as a difference smooth per level of the interaction between condition and day (i.e. a 3-way interaction) was estimated using functionality from the R package *mgcv* for fitting generalized Additive Models (GAMs)^22^. To also get parametric estimates of the effects involving the pitch index, we additionally fit a model approximating the effects of all continuous covariates as linear. To control for order effects, we included a population-level effect of learning session (1 or 2). We initially also accounted for potential confounding effects of gender with a binary and of age and time of day with smooth effects but dropped these factors due to low (explained) variability. Additionally, we included a monotonic effect of the number of foreign languages spoken and a smooth effect of years of musical training to capture parts of general ability traits potentially affecting vocabulary learning^23^. To test for effects of sleep duration on memory formation – separating intra- from inter-subject variability –, we fit two effects of self-reported hours of sleep in the nights preceding the training session, a smooth effect for the mean (“typical”) sleep duration per subject and a linear effect for the subject-centered variation (with a random slope), interacting with testing day. The full model specification is given as R-code with all *brms* calls in the supplemental information (Suppl. Formula 1, suppl. Table 1).

#### Motor influence on memory performance

To test for effects of cycling performance (average speed, speed variability (log CV), degree of synchronization (log IBD)) on memory recall, potentially interacting with stimulation condition and testing day (*hypothesis 3*), we fit three additional independent models, as including these metrics in the main model might have masked effects of pitch index, if motor performance were a mediator of any such effect. As IBD could only be measured in the isochronous stimulation condition, its estimates were copied to the respective observations in the self-initiated condition (because IBD could reflect a subject-specific trait – which in parts may have been driven by discrepancies between the stimulation rate and the individually preferred rate^24^. To improve numerical stability, we log-transformed CV and IBD but again estimated smooth non-linear effects of the three motor metrics, interacting with condition and day. As motor variability (CV) could reflect, both, general subject-specific characteristics and relative sensitivity to the stimulation condition, we again decomposed it into a subject-averaged (“between-subjects”) and a subject-centered (“within-subjects”) component. In these models, we controlled for effects of session, sleep duration (only linear effects), foreign languages and musical training in the same ways as in the pitch index model and included group-level (random) intercepts and slopes where appropriate.

#### Motor performance

To test for effects of pitch index on cycling/synchronization performance, potentially depending on the stimulation condition (*hypothesis 4*), we modeled average cycling speed with a Gaussian (identity link) and the CV and IBD with a Gamma likelihood (log link) whose conditional means *μ*_*i*_ were predicted by stimulation condition, pitch index, sex, session and the other control covariates included in the memory models. As speed and CV were observed twice per subject (once per stimulation condition), we again estimated the intercepts as randomly varying over subjects. In addition, we informed the speed model about the observed standard deviation (using the *se* response special term in *brms).* Capitalizing on the multivariate functionality implemented in *brms*, we fit all models of pitch index together (see supplemental information).

#### Prior specifications

We specified weakly informative priors on all population level and weakly regularizing priors on group-level and smoothness-controlling parameters, whereby the priors on the intercepts and on the effect of day on memory recall were data-derived to increase sampling efficiency (as we did not intend to make inference on these effects^25^). Details can be found in the extended methods section in the supplemental information.

We applied orthonormal contrast coding for all categorical predictors to achieve equal marginal priors on all pairwise differences between factor levels, so effects can be interpreted as classical main effects.

All analyses were performed in *R* 4.3.3 with *brms*^21^ using functionality of packages from the *tidyverse*^26^, *bayesplot*^27^ and *DHARMa* for model critique, *tidybayes*^28^ for calculating posterior marginal means and contrasts, and *ggdist*^29^ for visualization.

#### Model performance

All multivariate models (fit with 4 Markov chain Monte Carlo (MCMC) chains with 3000 iterations each) converged successfully (*Rhat* ≈ 1.00 and effective sample size ESS (bulk and tail) >  > 1000 for all parameters, indicating good chain mixing and reliable estimation). Posterior predictive checks indicated good out-of-sample predictive performance of the fit models, including the distributional characteristics *SD*, median and skewness that were not considered by the fitting algorithm. Residual analyses (e.g., QQ-plots) indicated no systematic remaining patterns or other problematic issues, except for a slight under-dispersion of the memory model, which implies that the estimated credible intervals are on the conservative side (i.e., possibly a bit too wide) and a few strongly influential data points (indicative of a slight over-fitting).

The Bayesian R^2^ of the memory models was around 0.84 ([2.5th, 97.5th] percentile: [0.79, 0.87], SE 0.019), of the speed model 0.53 [0.34, 0.68]), of the CV model 0.56 [0.37, 0.72], of the IBD model 0.83 [0.46, 0.98], of the effort model 0.51 [0.26, 0.69] and of the task-difficulty model 0.30 [0.15, 0.45]. Vocabulary test performance was consistent within subjects as indicated by a high intra-class correlation, ICC, (assessed via variance decomposition of the posterior predictive distribution, ranging between 0 and 1) of 0.56 [0.27, 0.72] (and 0.53 [0.22, 0.70] due to the random intercept alone) and a high correlation between the group-level intercepts of the pseudo and the polish vocabulary tests in the multivariate model (*rho* ≈ 0.57 [0.28, 0.79], pd ≈ 1.00). Pedaling speed (ICC 0.29 [-0.18, 0.58]) and variability (ICC 0.41 [− 0.31, 0.75]), as well as effort (ICC 0.36 [− 0.29 0.69]) and particularly task difficulty ratings (ICC 0.06 [− 0.38 0.38]) were less consistent across sessions. Out of the three motor metrics, CV was the best predictor of memory recall (see supplemental information).

#### Model evaluation and interpretation

In the following, we report and display posterior probability distributions of model parameters on the link (log-odds) scale (for simple linear effects and interactions), and marginal means of as well as contrasts between the expected values of the posterior predictions (i.e., the posterior fits) on the response scales for more complex interactions and non-linear (smooth) effects for better interpretability. For smooth effects, we also calculated the local (varying) slopes (i.e., the partial derivative of the posterior predictions with respect to a given continuous predictor) via the finite difference method. To summarize smooth effects, we additionally compared the average posterior predictions between the upper and the lower half of the given predictor (i.e., we performed median splits of the posterior model predictions). To quantify effect sizes and their estimation precision, we display the full posterior densities where possible and indicate the median and the 66%, 90% and 95% highest (continuous) density intervals (Bayesian credible intervals, CI), as the 95% interval tends to be numerically unstable^30^. The Bayesian credible intervals are summary metrics of a posterior distribution and contain the true effect with the respective probability; they should be distinguished from the frequentist confidence interval which is a long-run property of a procedure^31^. As an index of effect existence, we report the probability of direction (pd), which is the larger of the proportions of the posterior samples > or < 0, ranging between 0.5 and 1. The pd loosely corresponds to 1–0.5 times the two-sided p-value in a frequentist framework (i.e. a pd of 0.975 corresponds to a two-sided p-value of 0.05 32) and it is related to the posterior evidence ratio, ER, by ER = pd/(1–pd). In line with recommendations of the American Statistical Association^32–34^, we report unthresholded continuous probabilities. As a communicative device, we adopt the labels proposed by Jeffreys^35^ and Kass^36^ for Bayes factors in the context of model comparisons to communicate the graded evidence about an effect’s sign provided by the pd, which is justified when the prior odds are equal for θ > 0 and θ < 0, which is the case here. For the binomial memory model, we report the pd both on the link (i.e. log-odds) scale (e.g. the pd of the corresponding *β* parameter, if available), noted as pd_link_, as well as on the response scale (i.e. number of recalled items), calculated from the (marginal) contrasts of the model estimates and noted as pd_resp_. We do not report Bayes factors (BFs; Savage–Dickey density ratios) because no elicited alternative prior was available and BFs are biased towards the null hypothesis with the weakly informative regularizing priors used here (Bartlett–Lindley paradox^37,38^) and hence may be misleading.

## Results

### Memory performance

The participants recalled an average of 8.5 words (median 7, SD: 4.8) in the self-initiated condition and 9.4 (median 8, SD: 5.6) in the isochronous condition immediately after encoding on day 1. 24 h later, on day 2, they remembered on average 7.7 words (median 7, *SD*: 4.8) in the self-initiated condition and 8.3 (median 7, *SD*: 6.0) in the isochronous condition.

As expected, subjects recalled on average 0.9 [1.7, 0.0] (pd_resp_ ≈ 0.98) words less on the day following each learning session (6.4 [4.6, 8.3]) than immediately after training (7.2 [5.2, 9.1]; pd_link_ of the main effect of testing day on memory recall ≈ 1.00). In addition, we observed a consistent effect of session, with subjects remembering on average 1.6 [0.4, 2.8] words more in the second (7.6 [5.6, 9.8]) than in the first (6.0 [4.3, 7.8]) learning session (pd_link_ ≈ pd_resp_ ≈ 1.00).

### Influences of stimulation condition

Regarding *hypothesis 1*, we observed only weak evidence for a main effect of stimulation condition on recall performance (pd_link_ of the main effect of stimulation condition on memory recall ≈ 0.77; median 7.0 items, 95% CI [4.9, 9.1] in the isochronous compared to 6.5 [4.8, 8.5] items in the self-initiated stimulation condition, pd_resp_ of the contrast on the response scale ≈ 0.78), so we remain uncertain whether isochronous stimulation generally leads to better memory recall than self-initiated stimulation.

### Influences of pitch perception preference

**Fig. 2:**
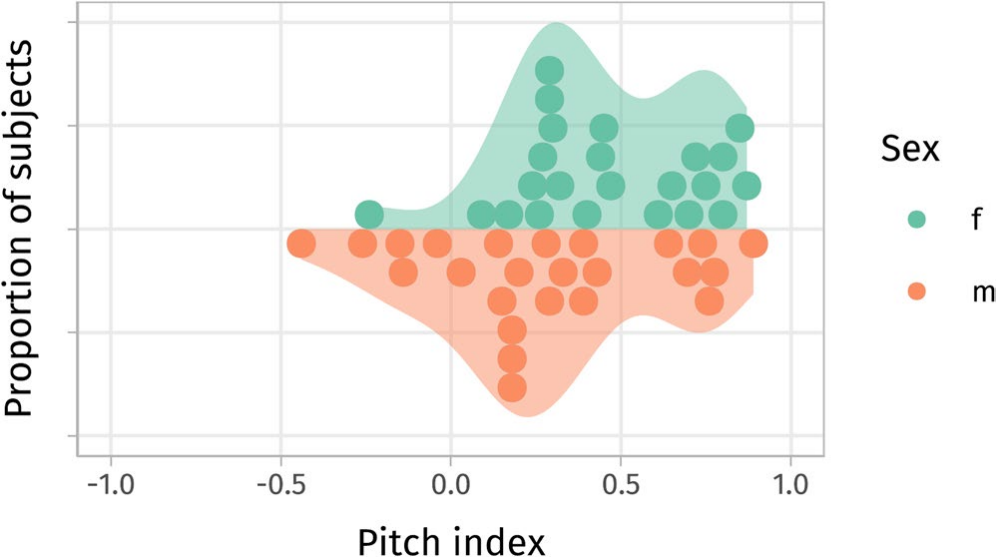
Distribution of the pitch perception index. The sample was biased towards SPPs and included no extreme FPPs (pitch index close to − 1). There was no systematic bias of sex and it was balanced between condition orders (see supp. Figure 1.)

The pitch perception index (PI) in our sample ranged between -0.44 and 0.89 (mean ≈ 0.36, median ≈ 0.32, SD ≈ 0.33), with a slightly left-skewed distribution (skewedness ≈ -0.30, see Fig. 2). While the low sampling density in the lower pitch index spectrum limited our ability to draw conclusions about (extreme) FPPs, it did not bias the results, as we handled pitch index as a continuous metric modeled with a smooth non-linear effect adapting to the evidence. There were slightly more male than female FPPs and pitch index was positively associated with years of musical training among subjects playing an instrument. It appears unlikely that this association is genuine in the light of other larger studies, and it is generally believed that pitch index is independent of musical training or aptitude (for details see supplemental information, suppl. Figure 2 an suppl. Table 2). Nevertheless, we controlled for any (coincidental) confounding effects of years of musical training (and number of foreign languages) in all statistical models, so the reported effects of pitch index are unbiased by this association (but possibly less precisely estimated).

**Fig. 3:**
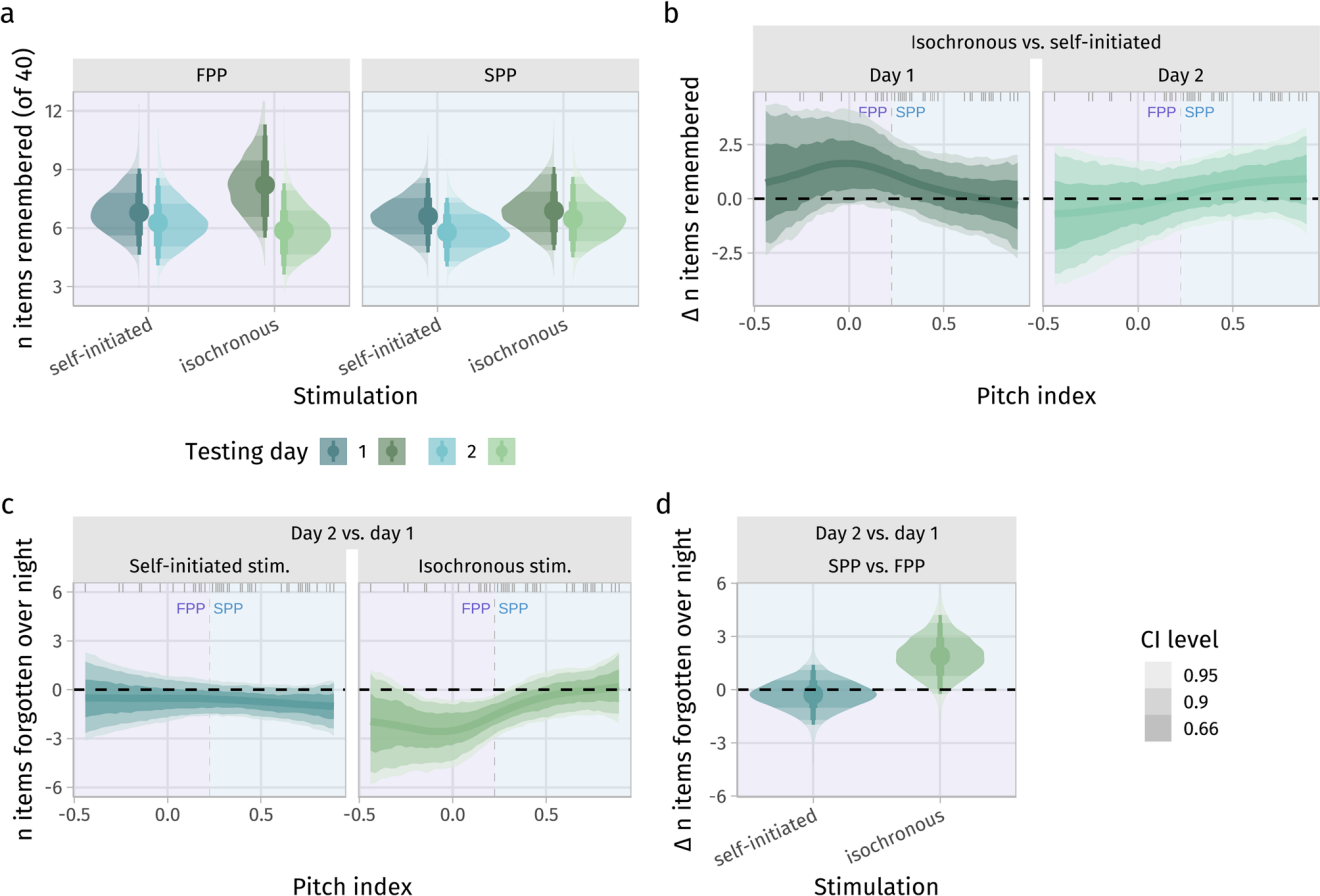
(**a**) Posterior distribution of the expected number of items remembered during the four vocabulary tests, with the smooth effect of pitch index split into two bins at the mean pitch index (vertical dashed lines in panels **b** and **c**) for illustrative purposes (left|right facet). Posterior distributions of the contrast between stimulation condition (**b**) and testing day (**c**) for the expected number of forgotten items overnight illustrate relatively greater forgetting (following a higher initial recall rate) after isochronous compared to self-initiated stimulation in FPPs and reduced forgetting in SPPs (see also suppl. Figure 3b). (**d**) summarizes the effect of pitch index by comparing the forgetting between the SPP and the FPP bin after either stimulation regime, showing that SPPs had better memory retention after isochronous stimulation compared to FPPs (see also suppl. Figure 3a for the local effects (slope) of pitch index). Lines/points are at the median of the posterior densities and lightness of the ribbons/slabs marks the 66%, 90% and 95% highest density credible intervals. Dashes on the top mark the observed pitch indices in the sample.

Regarding *hypothesis 2*, there was no simple interaction between stimulation condition and pitch index (max. pd_resp_ across a grid of 50 pitch index values, yielding an upper evidence bound of ≈ 0.79, pd_link,linear_ of the interaction term of the linear model ≈ 0.56, median ≈ 0.02). However, we observed moderate evidence for a 3-way interaction between pitch index, stimulation condition and testing day (pd_link,linear_ of the 3-way interaction term of the linear model ≈ 0.93, median ≈ 0.17; below, we describe the results of the non-linear model depicted in Fig. 3 and suppl. Figure 3). This interaction reflects that those subjects with a lower pitch index (more fundamental pitch perception, FPP) recalled on average 1.3 [− 0.9, 3.9] (pd_resp_ ≈ 0.88) more words (~ 8.3) directly after isochronous than after self-initiated stimulation (~ 7.0, see Fig. 3a). However, this advantage was not sustained over time, as these subjects remembered a roughly equal number of words as following self-initiated stimulation on the next day (~ 5.9 isochronous vs. ~ 6.4 self-initiated stimulation ≈ − 0.5 [range: − 2.7–1.7], pd_resp_ ≈ 0.67, see Fig. 3b). Concurrently, subjects with a higher pitch index (preference for spectral pitch perception, SPP) recalled slightly more items (0.6 [range: − 0.9–2.1], pd_resp_ ≈ 0.80) on the day following isochronous (~ 6.6) than on the day following self-initiated vocabulary presentation (~ 5.9), but showed no noteworthy difference on the first day (~ 6.9 isochronous vs. ~ 6.8 self-initiated stimulation ≈ 0.2 [range: − 1.3–1.7], pd_resp_ ≈ 0.61, see Fig. 3a + b). This can be expressed in terms of a slightly better (pd_resp_ ≈ 0.73, providing only weak evidence) vocabulary retention in SPPs after isochronous stimulation (0.4 [− 0.8, 1.4] items forgotten) compared to self-initiated stimulation (0.8 [− 0.2, 1.9] items forgotten) and greater forgetting in FPPs (pd_resp_ ≈ 0.93) , suggesting that the positive effect of regular stimulation was short-lived and limited to recall on day 1 (2.3 [range: 0.4–4.3] items forgotten).SPPs forgot 1.9 [range: − 0.3–4.2] fewer items after isochronous stimulation than FPPs (pd_resp_ ≈ 0.96, see Fig. 3c + d). In summary, we found moderate evidence for an improvement of short-term (but not long-term) recall by isochronous stimulation in FPPs, and a slight and relatively uncertain improvement of long-term recall in SPPs.

### Influences of cycling consistency

**Fig. 4:**
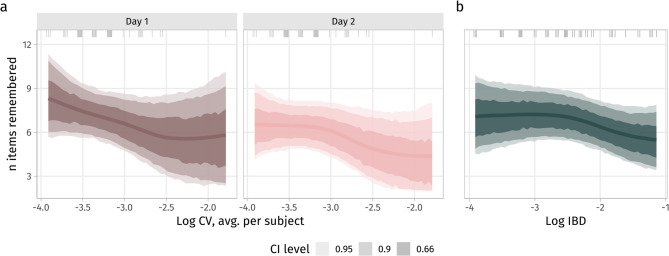
Recall performance was slightly better in subjects with lower cycling rate variability (log-transformed CV, a) and lower inter-beat deviation (log-transformed IBD, c), as indicated by a weakly negative slope over most of the observed log CV/IBD range (see partial derivatives in suppl. Figure 4). Dashes on the top mark the observed values. Note one subject with a very high CV (logarithmic scale).

Regarding *hypothesis 3*, we observed moderate evidence for positive effects of movement synchronization to isochronous stimulation (pd_resp_ of the mean partial derivative with respect to log IBD ≈ 0.87; pd_link,linear_ of the *β* coefficient of log IBD in the linear model ≈ 0.90, Fig. 4 b and suppl. Figure 4) as well as of average pedaling rate consistency (pd_resp_ of the mean partial derivative with respect to log CV ≈ 0.89, pd_link,linear_ of log CV ≈ 0.93, Fig. 4 a and suppl. Figure 4) on overall vocabulary recall performance, independent of (i.e. not interacting with) stimulation condition (assessed in separate models). There were no effects of average cycling speed on memory performance.

### Motor performance

Speed-normalized cadence variability (i.e., the CV) was on average lower during isochronous compared to self-initiated stimulation (pd_link_ ≈ 1.00, pd_resp_ ≈ 0.94), providing strong evidence for an influence of the stimulation regime in subjects of all musical experience levels. This implies that most subjects were aware of the sensorimotor relations and adjusted their behavior accordingly, although not explicitly instructed to synchronize the cycling cadence to the sounds.

Mean pedaling speed was on average 7 [2, 12] % (i.e., 0.07 Hz; pd_resp_ ≈ 1.00) above the target speed of 1 Hz implied by the (lead-in or ongoing) stimulation rate, 5 [− 1, 11] % (pd_resp_ ≈ 0.94) with self-initiated stimulation and even 10 [3, 16] % (pd_resp_ ≈ 1.00) with isochronous stimulation (AMS condition), a difference of 0.03 [− 0.02, 0.08] Hz (pd_resp_ ≈ 0.90, pd_link_ of the condition effect ≈ 0.93).

### Influences of pitch perception preference

**Fig. 5:**
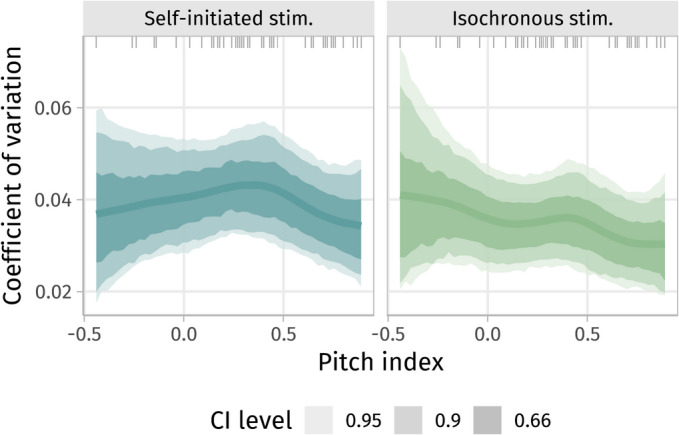
Relative cycling speed variability (CV) did not globally depend on pitch index, albeit there may be a slight trend of extreme SPPs to display lower variability than mixed pitch perceivers. (Note the low prevalence of FPPs (pitch index < 0) in the sample.) See also suppl. Figure 5.

Regarding *hypothesis 4*, there were no measurable systematic effects of pitch index (pd_link,linear_ ≈ 0.64, also no non-linear effects) on synchronization performance (IBD). There was weak to moderate evidence for a negative (i.e. opposite to the hypothesized) global relationship between pitch index and CV (pd_link,linear_ of a negative trend in the linear model ≈ 0.81, pd_link,linear_ of the interaction with stimulation condition ≈ 0.70). There was also a complex non-linear pattern that may indicate that strong SPPs had a lower intrinsic cycling variability (in the self-initiated condition) than subjects with a mixed pitch perception strategy (Fig. 5a), who, in turn, displayed a smaller CV in the isochronous compared to the self-initiated condition (Fig. 5b and suppl. Figure 5). While these effects are rather uncertain, as any trend (linear and non-linear) points into the opposite direction of our hypothesis (a lower intrinsic variability in FPPs in the self-initiated condition), we may reject it—albeit with low confidence, especially given the low number of (and absence of strong) FPPs in the sample. Pedaling speed was also independent of pitch perception preference.

### Influences of musical experience

**Fig. 6:**
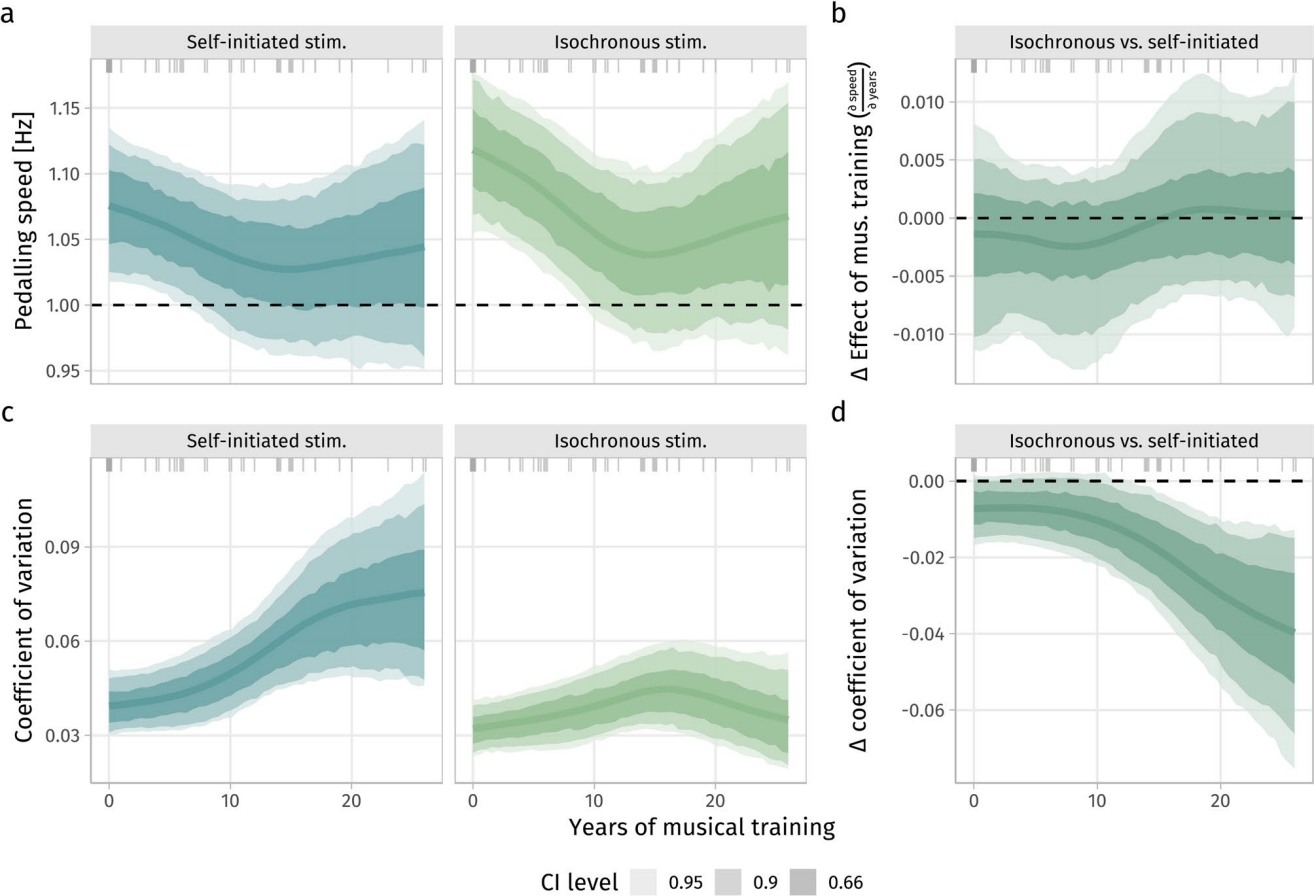
Mean pedaling speed (**a**) was above the implied target pace of 1 Hz (60 rpm), especially in subjects with less than 10 years of musical experience and even more so in the isochronous condition, where the stimulation rate was at a fixed rate of 0.5 Hz, leading to a slightly stronger effect (steeper partial derivative) of years of musical training on pedaling speed over the first 10–15 years in the isochronous compared to the self-initiated condition. This difference (Δ) in slopes (“local βs”), i.e. partial derivatives of speed w.r.t. years of musical training (δ speed/δ years) between the isochronous and the self-initiated condition is quantified in (**b**). Speed-normalized pedaling variability (CV) (**c**) increased with musical experience when pedaling triggered sound playback (left facet) but remained roughly equal over experience levels under isochronous stimulation (right facet). In subjects of all musical experience levels, isochronous stimulation was associated with lower pedaling variability compared to self-initiated stimulation (**c**), this difference being exacerbated by the years of playing a musical instrument (**d**). Note that CV is defined as SD(speed)/mean(speed), making CV and speed inversely related to each other; however, re-fitting the model with SD(speed) instead of CV yielded virtually the same picture.

Although no specific hypothesis was formulated regarding this relationship, we observed that the CV was more strongly associated with years of musical training, in a partly unexpected way: self-paced pedaling was more variable, the longer a subject played a musical instrument, but was approximately independent of musical training when synchronizing to the external isochronous sounds (Fig. 6c, pd_link,linear_ of a positive main effect in the linear model ≈ 1.00, pd_link,linear_ of a negative interaction with stimulation condition ≈ 0.98 due to the applied contrast coding, these effects can only be interpreted together, which is what Fig. 6c, d afford for the non-linear model). It seems that external stimulation strongly constrained intrinsic pedaling variability, having a more pronounced influence on the behavior of musically more experienced subjects.

Similarly, pedaling speed decreased over the first 10 years of musical training, approaching the implied target speed of 1 Hz (the pd_link,linear_ of the linear model ≈ 0.91 providing moderate to strong evidence for a negative effect), with this decrease being slightly more pronounced in the isochronous condition (Fig. 6a, b). No systematic effect of musical experience on the inter-beat deviation (IBD) could be detected (pd_link,linear_ ≈ 0.53, also no non-linear effects).

In addition, we observed that all three motor metrics were negatively associated with (average) sleep duration, indicating better synchronization in subjects sleeping more (see supplemental results and suppl. Figure 7).

## Discussion

In this study, we explored the relationship between auditory-motor coupling and vocabulary learning performance, focusing on inter-individual differences in auditory processing. To this end, we measured the pitch perception index^11^ as an indicator of sound and, likely, rhythm processing differences. We asked participants to learn new vocabulary under two distinct conditions: (a) an isochronous stimulation condition, where participants could synchronize their motor actions with the stimulus input, resulting in auditory-motor synchronization, and (b) a self-initiated condition, where participants moved at their preferred pace, with stimuli presented in alignment with their motor output.

Our findings partially confirmed our hypotheses. First, regarding *hypothesis 1*, evidence for an overall advantage of isochronous over self-initiated stimulus presentation for vocabulary learning was uncertain. Such an effect had been reported previously for stimulus encoding^6,8^ or predictive timing^39^.

However, we found moderate evidence in favor of *hypothesis 2,* as we observed a three-way interaction between stimulation condition, testing day and pitch index. Specifically, participants with a higher pitch perception index (more spectral pitch perception; SPPs) showed slightly better memory retention from day 1 (i.e., immediately after encoding) to day 2 (i.e., 24 h later) following isochronous compared to self-initiated stimulation. However, this partial pattern on its own should be regarded with low confidence. In contrast, individuals with a lower pitch perception index (more fundamental pitch perception, FPPs) displayed superior short-term recall performance in the isochronous condition on day 1 than SPPs. This benefit did not persist until the next day, appearing as a higher forgetting rate in this condition. However, it is important to note that participants with lower pitch perception preferences were underrepresented in our dataset. Furthermore, we found moderate evidence in favor of *hypothesis 3*, as recall performance was slightly better in subjects with better pedaling synchronization and lower pedaling variability (independent of pitch index).

We propose that distinct cognitive processes may underlie initial learning and memory consolidation and that the different sensorimotor contingencies in the experimental conditions may interfere with these processes differentially depending on individual auditory processing preferences. Although FPPs are thought to possess a neuroanatomical predisposition for fine-grained temporal processing—possibly due to a more strongly developed left primary auditory cortex^11,13,14^—our data showed that they did not outperform SPPs in terms of synchronization accuracy. This was in contrast to our *hypothesis 4*. In fact, strong SPPs exhibited slightly lower CV than mixed-mode perceivers though the effect was weak and conclusions about strong FPPs are hard to draw given their relatively small number and the absence of any strong FPPs in the sample. There was no direct relationship between pitch index and synchronization performance (IBD). Thus, *hypothesis 4* can be rejected with low to moderate confidence. This finding challenges the assumption that superior auditory temporal processing directly translates into better behavioral synchronization in the given task context. Nonetheless, FPPs may have coped better with the fixed and externally imposed vocabulary presentation rate, possibly finding the rhythmic structure more engaging or less disruptive. (Note that we did not directly test for a mediating role of motor performance in the effect of pitch perception preference on memory recall, as the total effect of pitch index was already rather uncertain, making it unlikely that a decomposition into a direct and an indirect path could be reasonably estimated.) The fixed rhythm may have provided a temporally structured context that aligned more naturally with the FPPs’ intrinsic processing preferences, thereby offering a (short-lived) motivational benefit. Supporting this interpretation, SPPs perceived the active AMS task as more challenging than FPPs (see supplemental information, suppl. Figure 8). It is therefore conceivable that fatigue or task-related demands accounted for the absence of any observable benefit of isochronous over self-initiated stimulation in SPPs during the initial learning session. In contrast, the enhanced engagement in FPPs could have facilitated early stages of memory encoding, leading to improved recall performance on day 1. This aligns with the finding that individuals who rated maintaining the cycling tempo as easier had slightly better vocabulary recall (see supplemental information). SPPs, on the other hand, may have benefited more from the consolidation of declarative memory—apparent in relative recall performance on the next day—, maybe because the more regular pedaling-independent (i.e., isochronous) stimulation may have been more salient fostering encoding. Isochronous stimulation possibly enhances the engagement and coordination of neuroanatomical pathways critical for memory formation and retrieval, such as prefrontal cortex and superior temporal gyrus^40^. Conversely, FPPs may rely less on external rhythmic cues because they have a higher intrinsic preference for rhythmicity, leading to a smaller benefit from AMS during the consolidation phase.

Importantly, our findings suggest that perceptual differences—presumably arising from variations in Heschl’s gyrus— may be associated with the extent to which participants benefit from isochronous stimulation. The hemispheric specialization for temporal (left) vs. spectral (right) auditory processing offers a useful framework for interpreting these findings. The left hemisphere’s ability to process rapid temporal sequences could enhance the (short-term or “live”) performance of FPPs in rhythmically demanding conditions^12^, while the right hemisphere’s specialization in spectral analysis may cause spectral pitch perceivers to profit from external rhythmic scaffolding for optimal memory consolidation. While we attempted to statistically control for confounding influences of auditory ability with years of musical training and of working memory or language ability with number of foreign languages as proxy metrics, we cannot entirely exclude the possibility that this relationship reflects broader shared factors rather than a direct influence of perceptual style itself which was a measured rather than a manipulated variable. Future studies could validate these neuroanatomical hypotheses using neuroimaging techniques to directly measure activity in Heschl’s gyri during AMS and self-initiated conditions.

Albeit not the focus of this study, we noted increased pedaling variability in the self-initiated condition among musically more experienced participants. This finding may reflect a heightened sensitivity to sensorimotor contingencies in this subgroup (see also supplemental information, suppl. Figure 9). This enhanced awareness might have enabled them to intentionally modulate their motor output to adapt the stimulus presentation rate to individual cognitive demands. Specifically, these participants may have strategically varied their pedaling speed to optimize encoding—for example, by slowing down during more complex or less familiar vocabulary items, thereby dynamically adjusting the temporal spacing of input based on perceived processing difficulty or momentary cognitive load. Such deliberate temporal control over stimulus presentation was only feasible in the self-initiated ‘feedback’ condition. In contrast, in the isochronous condition, where the temporal structure was externally imposed, these same individuals may have shifted to a strategy of stronger auditory–motor coupling, as also reflected in their reduced average pedaling speed, which was closer to the imposed target rate. Notably, across both conditions, musically experienced participants cycled more closely to the target speed than musically less experienced individuals, suggesting a more precise temporal alignment with the auditory stimuli irrespective of condition. This pattern further supports the idea that musical experience may facilitate the flexible use of motor behavior as a cognitive tool to optimize learning under varying task constraints^24,41–45^. Such an active exploitation of a detected sensorimotor contingency by more musically trained subjects may have mediated the observed performance increase in the vocabulary tests of these subjects, an effect that was additive to an advantage of experience with foreign languages (for both see supplemental information, suppl. Figure 6).

One limitation of the current study was that we did not include a seated condition for comparison with the auditory-motor coupling conditions. As such, we cannot definitively state whether both conditions are superior to sitting quietly and whether the advantage of musical training on vocabulary learning is of a general nature or specific to the audio-motor demands involved in both our conditions. However, when comparing our data to previous studies, performance in the seated condition was consistently lower than in both the AMS and self-initiated conditions (Schmidt-Kassow et al^5^: mean = 5.6 (*SD* = 3.0) words; Schmidt-Kassow et al.^4^: mean = 4.8 (*SD* = 4.2) words). Even though the actual superiority of both auditory-motor coupling conditions must be demonstrated in future (within-subject) experiments, the current findings suggest that both conditions may lead to an improvement. However, the specific benefit of each condition (AMS vs. self-initiated) depends on inter-individual differences in auditory processing. Future studies with similar paradigms should increase the variation width of such individual traits, for example by including also professional musicians and by actively seeking to sample pitch perception index more homogenously; in particular, sampling density on the lower side of the spectrum of pitch perception preferences (i.e., FPPs) was rather sparse in our dataset.

Taken together, our findings highlight the need to account for individual differences in auditory perception when assessing the cognitive effects of auditory–motor coupling. The interaction between motor engagement and auditory processing does not uniformly benefit all learners. In our sample, systematic inter-individual traits such as pitch perception mode, musical experience and synchronization performance modulated how participants responded to the different conditions. We also observed effects of factors that were not measured directly, such as general learning ability or strategy use, influencing the degree to which each auditory–motor regime affected memory formation. For example, overall vocabulary performance correlated with a memory benefit of the isochronous condition across individuals (see supplemental information, suppl. Figure 10). Future research should aim to disentangle these effects using a combination of behavioral, neurophysiological, and modeling approaches to better isolate auditory and motor contributions to learning.

## Supplementary Information

Below is the link to the electronic supplementary material.

Supplementary Material 1

## Data Availability

The datasets generated during the current study are available in the Github repository https://github.com/mschmidt-kassow/ams_learning.
