# Supplementary material for: Interindividual differences in auditory processing moderate the effect of auditory-motor coupling on paired-associate learning
Source: Sci Rep. 2025 Oct 17;15:36295. doi: 10.1038/s41598-025-23360-w (PMC12534385; doi:10.1038/s41598-025-23360-w)
Supplement: Supplementary file 1 — Supplementary Material 1 [file 41598_2025_23360_MOESM1_ESM.pdf]

Supplemental Information

Extended methods

Modeling perceived task difficulty. To test whether perceived "effort" or task difficulty ("ease of keeping the cycling speed") depended on the individual pitch preference, potentially conditional on the stimulation regime and the musical experience, we modeled these two 5-point Likert scale ratings with two cumulative probit models (multilevel ordinal regression), where the mean of a Gaussian distribution, interpretable as the latent cause of ratings, is conditional on the predictors, and 4 (common) thresholds are estimated to predict the discrete rating choices[1].

Multivariate modeling. Capitalizing on the multivariate functionality implemented in brms, we fit all models of pitch index (one memory model, three cycling performance models, 2 rating models and an additional model of hours of sleep used for imputing one missing value) together, estimating the group-level (i.e., random) effects (intercepts of speed, CV and ratings, and intercept as well as effects of condition and day on recall performance) as mutually correlated over subjects (i.e., as a multivariate Gaussian distribution). Besides increasing the fitting robustness, this allowed us to account and test for general "ability traits" (e.g., an overall better performance in both motor and memory tasks), as well as for a dependence between e.g., a condition effect on vocabulary recall and the average recall performance (the intercept) of a subject. In addition, we included a binomial model of recall performance in the pre-screening pseudo vocabulary test in this multivariate setup with correlated per-subject varying intercepts to increase fitting robustness. The full formula and prior specifications are represented as R-code below.

Details on prior specifications. On the intercept of the log-odds recall rate in the binomial memory models, we set a Student-t prior with 5 degrees of freedom (*df)* with the μ and σ parameters corresponding to the observed distribution of recall rates (parametrized by the empirical 50th and 90th percentile), corresponding to the default practice in *brms* for Gaussian and Gamma family models. Similarly, as we did not intend to make inference on the main effect of testing day and to increase sampling efficiency, we set the Student-t prior ($df = 5$) on the β parameter of day to correspond to the observed (and expected) negative recall differences between day 2 and day 1. On the β parameters of all other linear (scaled to roughly unit *SD*) predictors – including on the linear (null-space) components of the smooth effects – and on the

other binary predictors, we set weakly informative Student-t priors (*df* = 5) around 0 with the spreading parameter σ set to correspond to a ~ +/-50% change in recall rate per unit predictor change relative to the median observed recall rate of 7/40. As we expected a relatively high inter-individual recall variability, we set the σ of the positively bound and 0-centered Student-t priors on the *SD* of the group-level effects to twice the respective σ on the population-level effects (i.e. the βs) (*df* = 3 for the random intercept and *df* = 4 for the random slopes).

For the three cycling performance and the two rating models, we specified the priors for all βs (the numeric predictors again being scaled to ~ unit *SD*) as 5-*df* Student-t distributions centered at 0 with a σ of 1, except for the effect of stimulation condition, where we expected a larger effect on the motor metrics, reflected in a prior σ of 5. We set the priors on the intercept group-level *SD*s to 4-*df* half-Student-t distributions with μ = 0 and σ = 1, except for the observation-level random effect of IBD, where we applied more shrinkage by specifying *df* = 8 and σ = 0.5. Similarly, we specified stronger shrinking exponential(1) priors on the *SD*s of the rating random intercepts and on all random slopes.

To increase the anti-overfitting effect of the penalization structure of the smooth effects, we decreased the widths of the 0-centered half Student-t (*df* = 5) priors on the *SD*s of the smooth effects (acting as the inverse wiggliness penalization hyper-parameter λ in GAMs) to σ = 1.5 (relative to the *brms* default of 2.5).

The priors on the step sizes of the monotonic effect of number of foreign languages were left at their default, except for the step between 0 and 1 language, where we expected a larger effect than between e.g. 1 and 2, reflected in a dirichlet(2, 1, 1, ...) prior.

We specified the priors on the thresholds of the cumulative probit models as Gaussian distributions with σ = 1 and the respective μs distributed over equal quantiles of a standard Gaussian distribution.

The priors on all correlation matrices were left at the default Lewandowski-Kurowicka-Joe (LKJ) distribution with η = 1 (uniform).

R-code specification of the models investigating pitch index effects. Below, we print the code for specifying and fitting the Bayesian non-linear multivariate General Additive Mixed Model (GAMM) investigating the effects of pitch perception preference together with the effects of musical and language experience on behavior (memory performance, motor performance, perceived effort and perceived task ease). The three separate models for

investigating the effects of motor performance on memory and the respective linear versions (without s()-terms) are set up analogously and not shown here for reasons of brevity.

```
library(brms)

# ---------- Formulas ----------
# Notes:
#   All s() use thin-plate regression splines (bs = "tp"); only k (max. Df) is shown.
#   mo(): monotonic effect
#   subset(): use only a subset of the data to fit this model (e.g. only day1 for motor)

# Variable legend:
#   df_: data frame with scaled predictors
#   nRecall, nRecallPseudo: # of correct answers; nVocabs, nVocabs_pseudo: # of trials (vocabularies)
#   stim_cnd: stimulation condition (self/iso); day: testing day (day1/day2)
#   *_o: ordered versions to set up difference smooths (no smooth for the reference level)
#   cndXday: interaction of stim_cnd:day (ordered factor for difference smooths)
#   PI: pitch index; yearsMus: years of musical training; nForeignLang: number of foreign languages
#   hSleep_between: mean hours of sleep per subject; hSleep_within: subject-centered hours of sleep
#   session: session order (S1/S2); sex; subj: ID for random effects structure linked between models

# 1) Memory accuracy (binomial)
bf_recall <- bf(
  nRecall | trials(nVocabs) ~
    stim_cnd * day +
    s(PI, k = 10) + s(PI, by = cndXday, k = 10) +  # one common reference smooth + difference smooths
    session + s(hSleep_between, k = 7) +
    hSleep_within + hSleep_within:day +
    mo(nForeignLang) + s(yearsMus, k = 7) +
    (1 + stim_cnd + day + hSleep_within | p | subj),  # linked random effects via link-ID (| p |)
  family = binomial()
)

# 2) Pseudo-word pretest (binomial; only 1 value per subject from screening session)
# Inform model via correlated random effects (on link scale) about subject-specific memory performance
bf_recall_pseudo <- bf(
  nRecallPseudo | trials(nVocabs_pseudo) + subset(day1_iso) ~
    mo(nForeignLang) + (1 | p | subj),  # to estimate latent correlations on link-scale
  family = binomial()
)

# 3) Pedaling speed (Gaussian with known within-trial SD; day 1)
bf_speed <- bf(
  speed | resp_se(SDspeed, sigma = TRUE) + subset(day1) ~
    stim_cnd +
    s(PI, k = 10) + s(PI, by = stim_cnd_o, k = 10) +
    session + sex + s(hSleep_between, k = 7) +
    hSleep_within +
    s(yearsMus, k = 7) + s(yearsMus, by = stim_cnd_o, k = 7) +
    (1 + hSleep_within | p | subj),
  family = gaussian()
)

# 4) Speed-normalized pedaling variability (CV; Gamma-log; day 1)  — same predictors as bf_speed
bf_cv <- bf(
  CV | subset(day1) ~
    stim_cnd +
    s(PI, k = 10) + s(PI, by = stim_cnd_o, k = 10) +
    session + sex + s(hSleep_between, k = 7) +
    hSleep_within +
    s(yearsMus, k = 7) + s(yearsMus, by = stim_cnd_o, k = 7) +
```

```
    (1 + hSleep_within | p | subj),
  family = Gamma(link = "log")
)


# 5) Inter-beat deviations (IBD; Gamma-log; day 1 iso only)
bf_ibd <- bf(
  IBD | subset(day1_iso) ~
    s(PI, k = 8) + session + sex +
    s(hSleep, k = 7) + s(yearsMus, k = 7) +
    (1 | p | subj),
  family = Gamma(link = "log")
)


# 6 & 7) Ratings (cumulative-probit; day 1) — effort and ease share the same predictors
# Do SPPs perceive the iso condition as more difficult compared to the self than FPPs?
# (potentially driven by awareness + performance effects)
rating_rhs <- ~ stim_cnd +
  s(PI, k = 8) + s(PI, by = stim_cnd_o, k = 8) +
  session + sex +
  s(yearsMus, k = 7) + s(yearsMus, by = stim_cnd_o, k = 7) +  # may increase awareness or performance
  (1 | p | subj)  # captures individuals making different use of the scale


bf_effort <- bf(effort | subset(day1)           ~ rating_rhs, family = cumulative(link = "probit"))
bf_ease   <- bf(easeSpeedKeeping | subset(day1) ~ rating_rhs, family = cumulative(link = "probit"))



# ---- Prior helpers (binomial logit & ordinal thresholds) ----


#' Map probability beliefs to Normal(mu, sd) on logit scale using two quantiles (50% & 90%).
#' q50: median p; q90: 90th-percentile p (belief upper bound).
#' Returns c(mu, sd) for normal prior on logit(p).
probs2musigma <- function(q50, q90) {
  beta_params <- ProbBayes::beta.select(list(x = q50, p = 0.5),
                                        list(x = q90, p = 0.9))
  p_sim <- rbeta(10000, beta_params[[1]], beta_params[[2]])
  theta <- qlogis(p_sim)
  c(mean(theta), sd(theta))
}


#' Build threshold priors for cumulative-probit with flexible thresholds:
#' intercept_i ~ Normal(qnorm(i/n), sd_thresh)
make_threshold_priors <- function(df, resp, sd_thresh = 1) {
  n <- nlevels(as.factor(df[[resp]]))
  if (n < 2) stop("Need at least two ordered levels.")
  pri <- lapply(seq_len(n - 1), function(i) {
    set_prior(sprintf("normal(%.3f, %.3f)", qnorm(i / n), sd_thresh),
              class = "Intercept", coef = i, resp = resp)
  })
  do.call(c, pri)
}


# ---- Data-driven summaries for binomial intercepts (logit scale) ----
# Memory accuracy (main task)
p_recall_med  <- median(df_$nRecall / df_$nVocabs, na.rm = TRUE)
p_recall_q90  <- quantile(df_$nRecall / df_$nVocabs, 0.90, na.rm = TRUE, names = FALSE)
tmp           <- probs2musigma(p_recall_med, p_recall_q90)
prior_recall_mu <- tmp[1]
prior_recall_sd <- tmp[2]


# Pseudo-word pretest
ix_iso <- isTRUE(df_$day1_iso)  # day1_iso subset
p_pseudo_med <- median(df_$nRecallPseudo[ix_iso] / df_$nVocabs_pseudo[ix_iso], na.rm = TRUE)
```

```r
p_pseudo_q90 <-  quantile(df_$nRecallPseudo[ix_iso] / df_$nVocabs_pseudo[ix_iso], 0.90, na.rm = TRUE,
names = FALSE)
tmp2             <- probs2musigma(p_pseudo_med, p_pseudo_q90)
prior_pseudo_mu  <- tmp2[1]
prior_pseudo_sd  <- tmp2[2]


# Day contrast prior (nudged negative): use subject-averaged day difference to set center & scale
# on logit.
# Compute per-subject mean change day2 - day1 in recall proportion; then median & 90th percentile
# of the change.
day_diff_by_subj <- df_ |>
  dplyr::group_by(subj, stim_cnd) |>
  dplyr::arrange(day, .by_group = TRUE) |>
  dplyr::summarize(diff = diff(nRecall / nVocabs), .groups = "drop") |>
  dplyr::summarize(diff = mean(diff), .groups = "drop")


day_med  <- stats::median(day_diff_by_subj$diff, na.rm = TRUE)
day_q90  <- stats::quantile(day_diff_by_subj$diff, 0.90, na.rm = TRUE, names = FALSE)


# Map a symmetric change ±(day_med, day_q90) around baseline p_recall_med to logit center/sd for
# the day contrast.
# Contrast coding (~ ±0.5) implies half-difference around the baseline.
prior_day_mu <- qlogis(p_recall_med - 0.5 * day_med) - qlogis(p_recall_med + 0.5 * day_med)
prior_day_sd <- abs(
  (qlogis(p_recall_med - 0.5 * day_q90) - qlogis(p_recall_med + 0.5 * day_q90)) - prior_day_mu
)


# Slope scale for binomial betas (~ “±50% around median” on odds/probability scale)
# Convert two probability levels (baseline and baseline+delta) to an sd on the logit scale.
sigma_b <- probs2musigma(p_recall_med, min(0.999, 1.5 * p_recall_med))[2]


# Monotonic effect levels
n_lev_nForeignLang <- max(df_$nForeignLang, na.rm = TRUE) + 1


# Ordinal thresholds priors (effort, ease), sd = 1
priors_thresh <- do.call(c, lapply(c("effort", "easeSpeedKeeping"),
                                   make_threshold_priors, df = df_, sd_thresh = 1))


# ---------- Define Priors ----------
# Set data-derived intercepts explicitly for binomial & Gamma family; others use the brms
# data-derived defaults
priors <- c(
  # Binomial intercepts (centered at observed medians on logit scale)
  prior(student_t(5, prior_recall_mu,  prior_recall_sd),  class = Intercept, resp = "nRecall"),
  prior(student_t(5, prior_pseudo_mu,  prior_pseudo_sd),  class = Intercept, resp = "nRecallPseudo"),
  # Note: To be able to use variables in the prior-specifications, we actually used:
  # set_prior(glue("student_t(5, {prior_recall_mu}, {prior_recall_sd})"), class = ...)


  # Binomial slopes
  prior(student_t(5, 0, sigma_b), class = b, resp = c("nRecall","nRecallPseudo")),
  # Day effect (contrast); nudged negative via prior_day_mu (no inference made on day)
  prior(student_t(5, prior_day_mu, prior_day_sd), class = b, coef = "day2M1", resp = "nRecall"),


  # Motor outcomes: weakly informative slopes around 0
  prior(student_t(5, 0, 1), class = b, resp = c("speed","CV","IBD")),
  prior(student_t(5, 0, 5), class = b, coef = "stim_cndisoMself", resp = c("speed","CV")),  # larger SD


  # Ratings: mildly regularizing betas; flexible thresholds
  prior(normal(0, 1), class = b, resp = c("effort","easeSpeedKeeping")),
  priors_thresh,
```

```
  # Group-level SDs (interpretation: ~90% of expected changes within ±50% in log-odds;
  # Gamma-log ≈ ×/÷2)
  prior(student_t(4, 0, 2 * sigma_b), class = sd, group = "subj", resp = "nRecall", lb = 0),
  prior(student_t(3, 0, 2 * prior_recall_sd), class = sd, coef = "Intercept", group = "subj"),
  prior(exponential(1), class = sd, coef = "hSleep_within", group = "subj", resp = "nRecall"),

  prior(student_t(4, 0, 1), class = sd, group = "subj", resp = c("speed","CV"), lb = 0),
  prior(exponential(2), class = sd, coef = "hSleep_within", group = "subj", resp = c("speed","CV")),
  prior(student_t(8, 0, 0.5), class = sd, group = "subj", resp = "IBD", lb = 0),
  prior(exponential(1), class = sd, group = "subj", resp = c("effort","easeSpeedKeeping"), lb = 0),

  # Smoothness SDs (slightly stronger than brms default)
  prior(
    student_t(5, 0, 1.5), class = sds,
    resp = c("nRecall","speed","CV","IBD","effort","easeSpeedKeeping"), lb = 0
  ),

  # Monotonic effect: Dirichlet on step sizes
  prior(dirichlet(c(2, rep(1, n_lev_nForeignLang - 2))), class = simo,
        resp = c("nRecall","nRecallPseudo"), coef = "monForeignLang1")
)

# ---------- Fit (compact) ----------
fit <- brm(
  bf_recall + bf_recall_pseudo + bf_speed + bf_cv + bf_ibd + bf_effort + bf_ease + set_rescor(FALSE),
  data = df_, prior = priors,
  chains = 4, iter = 3000,
  control = list(adapt_delta = 0.99, max_treedepth = 10),
  backend = "cmdstanr", seed = 42, cores = 4
)
```

Suppl. Formula 1. Compact code representation of the Bayesian multivariate GAMM investigating effects of pitch index together with musical and foreign language experience on memory and motor performance as well as on subjectively perceived effort and task ease.

For binomial/logit, the chosen SD scales imply ≈90% of expected subject-level variation lies within ±50% change around the median odds; for Gamma–log they imply about a halving/doubling on the response scale.

Model comparison. Leave-one-out (LOO) model comparisons using Pareto-smoothed importance sampling (PSIS-LOO; [2]) indicated that allowing for smooth non-linear effects of continuous predictors did not improve out-of-sample predictive accuracy of memory recall (expected log predictive density, ELPD, over held-out samples of the linear model = -448.3 (SE = 10.0) and of the non-linear model = -448.36 (SE = 9.9), difference = -0.07, SE = 1.26), but that inclusion of the smooth effects did also hardly increase model complexity due to the penalization (p_loo of the linear model = 53.9 (+/- 5.0) vs. 55.0 (+/-5.2) of the non-linear model), justifying to report the smooth effects for inferential purposes. Out of the three motor metrics, CV (with a between- and a within-subject component) was the best predictor of memory recall, as indicated by a difference in ELPD between the (non-linear) IBD and speed models compared to the CV model of -2.31 (SE 1.78) and -2.84 (SE 1.97), respectively.

Comparing the (non-linear and linear) memory models with and without pitch index in the predictor terms (including all interactions) with PSIS-LOO, we found no improvement, but also no (or only negligible) worsening, of out-of-sample predictive accuracy by including pitch index in any of the models (|ΔELPD| < SE of ELPD), except for IBD (ΔELPD ≈ -6.49, SE ≈ 0.81) and the rating of ease of keeping the speed (ΔELPD ≈ -1.76, SE ≈ 0.80), for which the simpler models performed significantly better (details see suppl. Table 1). In the case of IBD, this is explainable by the fact that only half the data were available to estimate any effect as it was only defined in the isochronous condition; consequently, no effect of pitch index was detectable in the model including it. Also, the Bayesian R^2 [3] did not differ above the size of random numerical fluctuations. However, as the pitch index is of substantive theoretical interest and we wished to estimate its posterior effect size directly, it is justified to retain it in the explanatory models [4,5]. Likewise, including control variables such as years of musical training and number of foreign languages independently of any improvement in predictive accuracy is motivated by the fact that omitting theoretically relevant covariates can induce omitted-variable bias in the estimation of other effects[6]. We therefore payed an estimation precision price (increased CIs) for obtaining more valid and interpretable estimates of the focal relationships. Our uncertainty estimates are thus rather on the conservative side.

**Response var**	**Better model**	**Δelpd**	**SE**	**p**	**|Δelpd|>2×SE**
nRecall	w PI ≈ w/o PI	-0.33	2.69	0.45	
speed	w/o PI	-0.94	1.08	0.19	
CV	w/o PI	-1.43	1.75	0.21	
IBD	w/o PI	-6.49	0.81	<.001	*
effort	with PI	+0.81	0.74	0.14	.
easeSpeedKeeping	w/o PI	-1.76	0.80	0.01	*

Suppl. Table 1. PSIS-LOO results of comparing the multivariate GAMM model with pitch index (w PI) and without pitch index (w/o PI) in the predictor terms, for every response variable. For the main response of interest, number of recalled vocabularies (nRecall), there was no notable difference, so including pitch index was neutral with regard to the model's out-of-sample predictive accuracy (expected log predictive density, ELPD). Similarly, predictive accuracy was negligibly affected by including pitch index in the models of speed and CV (|ΔELPD| < standard error (SE) of the ELPD). Only for IBD (estimated on half the data) and the rating of the ease of keeping the speed, the average log probability of held-out observations was significantly lower when including pitch index (|ΔELPD| < 2 x SE), indicating overfitting. Effort predictions slightly profited from including pitch index (|ΔELPD| < SE).

Supplemental results

Distribution of pitch index and associations with other subject characteristics

The pitch perception index (PI) in our sample ranged between -0.44 and 0.89 (mean ≈ 0.36, median ≈ 0.32, SD ≈ 0.33), with a slightly left-skewed distribution (skewedness ≈ -0.296). There were no strong FPPs with a pitch index below -0.44. There was no association with age ($r \approx 0.09$, $p \approx 0.5$), but there were slightly more male FPPs than females (Δ pitch index ≈ -0.18, $t_{45} \approx 0.06$).

There was a positive association between pitch index and years of musical training in our sample meaning more spectral pitch perception among subjects with longer instrumental history. The overall Pearson correlation coefficient was $r \approx 0.27$ [-0.01, 0.52], $p \approx 0.06$ (0.09 with Benjamini–Hochber FDR correction for the multiple comparisons displayed in suppl. Fig. 2)). When fitting a Bayesian negative binomial hurdle model of years of musical training, we found strong evidence for an association between years of musical training and pitch index (median beta ≈ 0.54 95% CrI [-0.02, 1.12], pd ≈ 0.96), but only among subjects playing an instrument, whereas the hurdle component, capturing the step to start playing an instrument, was independent of pitch index (pd ≈ 0.53).

While interesting, this finding should probably be interpreted with caution given the relatively low sample size, especially in the light of previous studies with much larger sample sizes. Schneider et al. [7,8] did not find any statistically significant associations between musicianship and pitch index in a large sample comprising 334 professional and 75 amateur musicians as well as 54 non-musicians. The same was reported for an independent dataset of 420 amateur and non-musicians by Ladd et al.[9]. On the other hand, Seither-Preisler et al.[10] found *more* predominant fundamental pitch perception among musicians compared to non-musicians, albeit with a quite different test design. A recent long-term study by Schneider et al.[11] found a modest increase in fundamental pitch perception prevalence among "musician" compared to non-musician children and adolescents (aged 7 to 19), mirrored in a negative correlation between a musical aptitude score (AMMA test) and pitch index at age 16; however, in two separate adult datasets (from Bücher et al.[12]) included in that study, the pattern appears reversed or insignificant. In contrast, an increase of fundamental pitch perception prevalence with development over childhood and adolescence appears more robust in the longitudinal study; but Ladd et al.[9] found the opposite pattern in the cross-sectional study with adults.

In summary, there is no consistent evidence for a relationship between musical training or aptitude and pitch perception preference. In general, it is believed that pitch index and cortical asymmetry of HG are independent of musical aptitude or training, but that absolute cortical thickness and electrophysiological markers such as P1 latency etc. are strongly related with musicianship (Schneider et al. [8,11]).

Biases of pitch index in datasets may arise due to clustering of certain types of pitch perceivers e.g. in musical ensembles (e.g. if subjects were recruited primarily from one or just a few orchestra(s)) or musical instruments (e.g. if subjects were recruited via their teachers etc.; cf. [7,13])

It may be worthwhile to re-analyze all available datasets (e.g. the ones mentioned above together with our own datasets) in one hurdle model with an appropriate random-effects structure (including random slopes) to more conclusively answer the question of a relation between musicianship/musical aptitude and pitch perception preference.

For the purpose of our study, this (possibly coincidental) association between pitch index and musical experience could not have biased the reported effects of pitch index, as we controlled for (potentially non-linear) effects of years of musical training in all models, i.e. we controlled for the potential confounding (or mediating) component of musical training. There were also no multi-concurvity (the non-linear generalization of colinearity) issues. Colinearity does generally not bias results, but could decrease estimation precision (wider CIs) or lead to identifiability problems, apparent in convergence issues or unstable fits; as all models successfully converged with sufficiently large effective samples sizes (ESS >> 1000) (and the regularizing priors prevented identifiability problems), that was no concern in our analyses.

In our experiment, pitch index was homogeneously distributed across both stimulation condition orders (median pitch index 0.29 and 0.33, mean 0.36 and 0.37, SD 0.35 and 0.31, for the isochronous stimulation first or second, respectively; t_{45} of the difference ≈ 0.125, p ≈ 0.9; see suppl. Fig. 1).

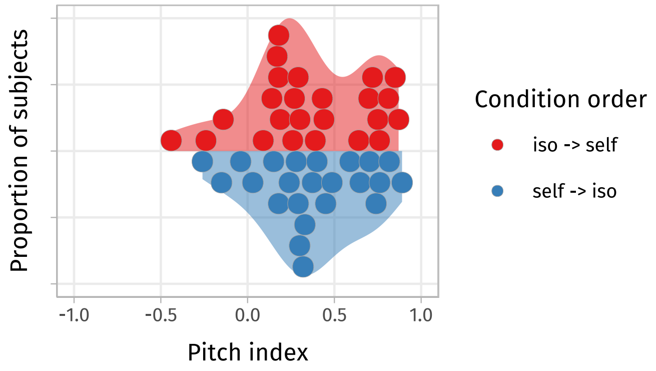

Suppl. Figure 1. Pitch index was equally distributed between the two orders of stimulation condition, first isochronous then self-initiated in red and first self-initiated then isochronous in blue.

Associations between other subject characteristics

Correlations between various various subject characteristics are depicted in suppl. Fig. 2 (with asterisks indicating uncorrected p-values < 0.05) and in suppl. Table 2 (with FDR-corrected p-values according to Benjamini & Hochberg, 1995).

Weak negative correlations between age and years of musical training and between age and number of foreign languages are likely spurious, given the low variance of age (SD = 2.8 years), the counter-intuitive negative sign and the relatively uncertain multiple comparison-corrected p-value of 0.09. In any case, as age did not show any detectable relation with any of the outcome variables of interest in this study (and was therefore dropped from the models) and had very low variance, it is unlikely that age confounded any of the results.

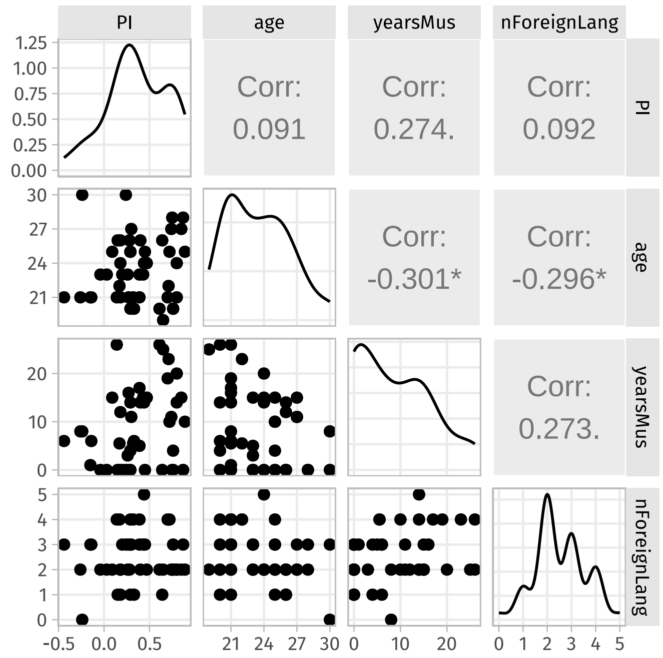

Suppl. Figure 2. Associations between subject characteristics. The scatter plots in the lower left triangle display the variables in the rows (y-axis) vs. the variables in the columns (x-axis). The upper right triangle shows the corresponding Pearson correlation coefficients, r, with the asterisks indicating uncorrected p-values below 0.05. The diagonal depicts the marginal distributions as kernel density estimates. PI: pitch index, yearsMus: years of musical training, nForeignLang: number of foreign languages spoken. There was a weak positive correlation between pitch index and years of musical training (see text for a more thorough analysis by means of a hurdle regression) and a (likely spurious) weak negative correlation between age and years of musical training and between age and number of foreign languages.

Pair	r	95% CI	t(45)	p
PI – age	0.09	[−0.20, 0.37]	0.61	0.54
PI – yearsMus	0.27	[−0.01, 0.52]	1.91	0.09
PI – nForeignLang	0.09	[−0.20, 0.37]	0.62	0.54
age – yearsMus	−0.30	[−0.54, −0.02]	−2.12	0.09
age – nForeignLang	−0.30	[−0.54, −0.01]	−2.08	0.09
yearsMus – nForeignLang	0.27	[−0.02, 0.52]	1.91	0.09

Suppl. Table 2. Pearson correlations between subject characteristics. FDR p-value adjustment by Benjamini & Hochberg (1995).

Additional Figures relating to the main results

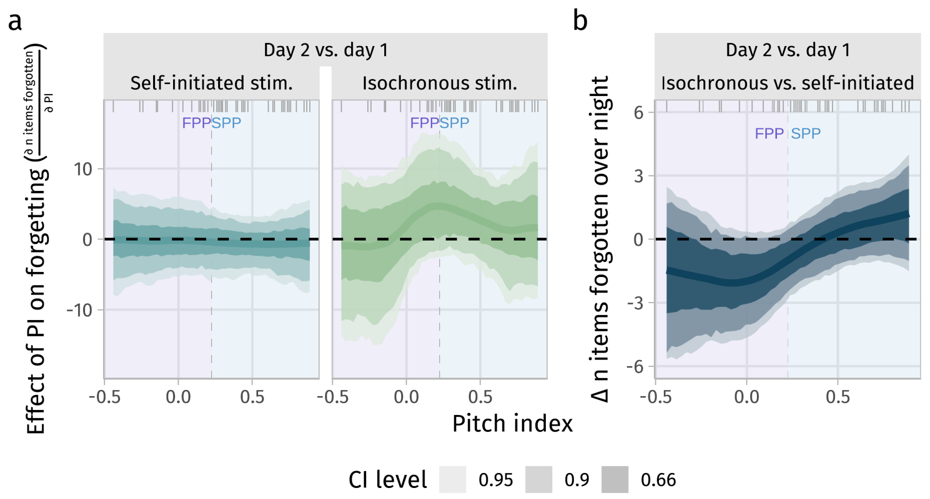

Suppl. Figure 3, relating to Fig. 3 in the main paper. a) displays the local effect (slope) of the pitch index on the number of items forgotten over night (calculated as the partial derivative of the number of items forgotten w.r.t the pitch index) varying over the range of observed pitch indices. b) shows the contrast between testing days of the difference in the number of recalled words between stimulation conditions (i.e. the difference of a difference), illustrating the 3-way interaction of relatively more forgetting (following a higher initial recall rate) after isochronous compared to self-initiated stimulation in FPPs and less forgetting in SPPs.

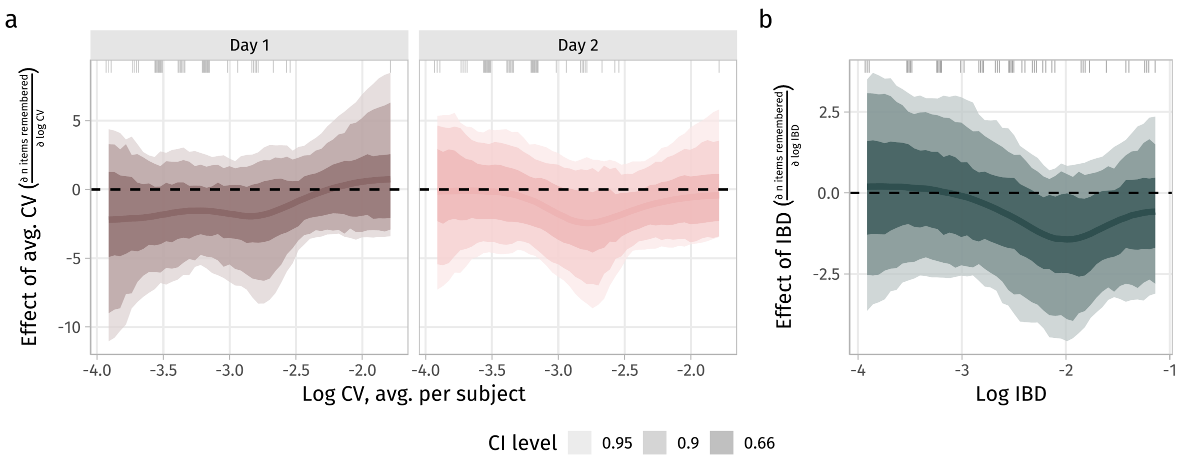

Suppl. Figure 4, relating to Fig. 4 in the main paper, displaying the partial derivative of the expected recall performance w.r.t. to cycling variability (a) and synchronization degree (b) as a function of these covariates. Recall performance was slightly better in subjects with lower cycling rate variability (log-transformed CV, a)) and lower inter-beat deviation (log-transformed IBD, c)) as indicated by a weakly negative slope over most of the observed log CV/IBD range.

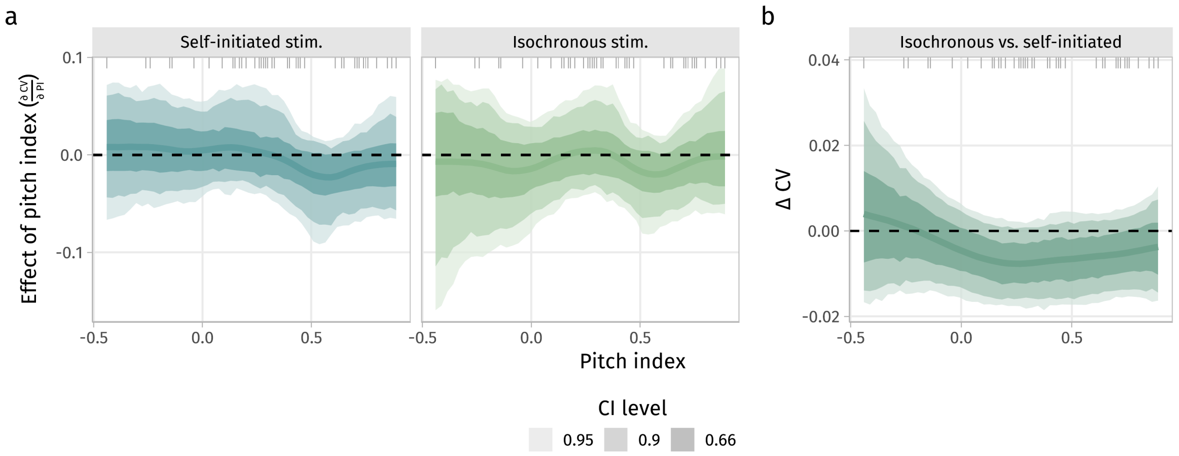

Suppl. Figure 5, relating to Fig. 5 in the main paper. There was no clear global trend of a dependence of relative cycling variability (CV) on pitch index, as indicated by the slopes (local effects of pitch index) fluctuating around 0 (a). CV was generally lower with isochronous than with self-initiated stimulation, except in the few true FPPs in the sample (pitch index < 0) who exhibited slightly higher CV in the isochronous condition, but due to the low sampling density in this region no general conclusion can be drawn from this dependence.

Influences of language and musical experience on vocabulary learning

We observed that recall performance in the Polish (but not in the German pseudo words) vocabulary tests increased with the number of foreign languages spoken (pd_{link} of an average positive slope of the monotonic effect ≈ 0.98, BF ≈ 2.2; suppl. Fig. 6a) as well as with the years of musical training ($pd_{link,linear}$ of the linear model ≈ 0.98, BF_{linear} ≈ 2.1; $pd_{resp,GAM}$ of the average partial derivative ≈ 0.95; suppl. Fig. 6b and c).

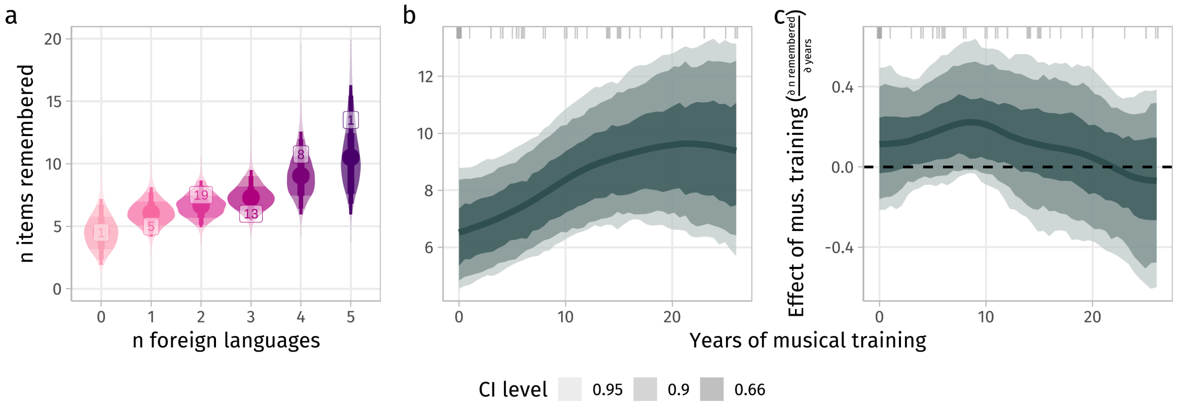

Suppl. Figure 6. Subjects speaking more foreign languages (a) and playing a musical instrument for a longer time (b and c) performed better in the Polish vocabulary tests. Numbers in (a) (positioned at the respective observed medians) signify the number of subjects in each category and the dashes on the top in (b) and (c) mark the individual observations (with some jitter).

Influences of sleep on memory and cycling performance

Sleep duration before the training sessions affected both memory recall and motor performance.

Subjects with a higher average sleep duration pedaled slower (approaching the implied target speed of 1 Hz; $pd_{link,linear}$ of a fitted linear effect ≈ 0.99, BF ≈ 4.0), synchronized their cycling cadence more strongly to the isochronous sounds (lower IBD; $pd_{link,linear}$ of the linear effect of average sleep duration per subject ≈ 0.99, BF ≈ 1.5) and varied less in their pedaling speed (lower CV, $pd_{link,linear}$ ≈ 0.94, BF ≈ 0.2), all three summary statistics likely reflecting the same underlying process, as they were derived from the same data. Suppl. Fig. 7 (b-d) displays the fitted smooth effects of the GAM models. Note that the effects of sleep duration and of musical training were estimated together in the same model(s), making it unlikely that they by chance reflect the same underlying causal effect.

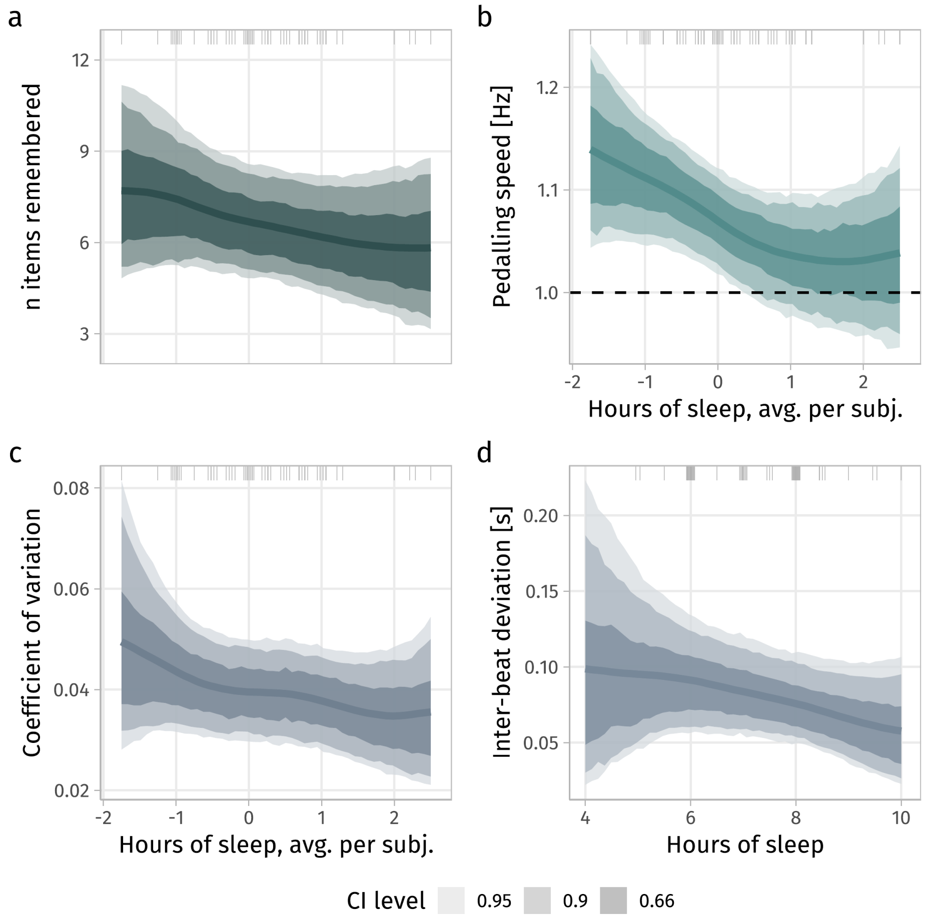

Suppl. Figure 7. Vocabulary recall (a), pedaling speed (b), pedaling variability (c) and inter-beat deviation (d) all decreased with the average hours of sleep per subject. The negative dependence of memory was the most uncertain, while the dependence of pedaling speed and IBD were robust.

The association between memory performance and sleep duration remained less conclusive. While average sleep duration was slightly *negatively* associated with memory recall ($pd_{link} \approx 0.85 - 0.93$, BF ≈ 0.4-0.7 over the 4 different memory models, one focusing on effects of pitch index, the other three on the motor indices; suppl. Fig. 7a), within-subject variance in sleep duration was slightly *positively* associated with average memory recall ($pd_{link} \approx 0.74 - 0.79$, BF ≈ 0.2) and with recall performance on the next day ($pd_{link} \approx 0.79 - 0.84$, BF ≈ 0.3). Note that as there were only two sleep-measures per subject, the estimation of the effect of within-subject variability in sleep duration is expected to be imprecise but should not be biased or bias the orthogonal between-subject effect, as we included a random slope of it.

Perceived effort and task difficulty

Noting the double dissociation between testing day and pitch index on the effect of stimulation regime on vocabulary recall, characterized by FPPs exhibiting improved intermediate-term and SPPs slightly improved long-term memory (but no effect on intermediate-term memory), we hypothesized that SPPs may have found the rhythmic audio-motor coordination tasks – particularly the AMS (isochronous) condition – more challenging (and hence fatiguing), leading to interference with memory *recall* on the day of the training, potentially masking any underlying beneficial effect of the AMS condition on memory *encoding*. We therefore tested whether self-reported task "effort" and "ease of keeping the cycling speed" depended on the pitch perception preference, potentially conditional on the stimulation regime and musical experience, by means of two cumulative probit models (one per question), within the multivariate model focusing on effects of pitch index.

We indeed observed a weak trend of subjects with more dominant spectral pitch perception (SPPs) to rate in particular the isochronous (AMS) condition as more effortful and less easy than subjects who relied more on rate pitch (FPPs; suppl. Fig. 8a and d, as well as c and f for the binned contrast; pd_{resp} of the binned contrast ≈ 0.69 in the self-initiated and 0.74 in the isochronous and condition). All subjects found it more challenging to "keep the cycling speed" with isochronous compared to self-initiated stimulation (likely indicating awareness of the AMS task; $pd_{link} \approx 0.88$, BF ≈ 0.5; suppl. Fig. 8b), while FPPs rated the effort (likely reflecting perception of physical demands) during isochronous stimulation as lower than during self-initiated stimulation (suppl. Fig. 8e). However, while congruent with our expectation based on the pattern in memory recall performance, these effects are rather uncertain and do not provide conclusive evidence on their own.

Compared to these subtle effects of pitch preference, session and sex had more consistent effects on ratings: Subjects consistently rated the second session as more effortful than the first one ($pd_{link} \approx 0.98$, BF ≈ 2.9) and male subjects found the tasks easier than female subjects ($pd_{link} \approx 1.00$, BF ≈ 26.5), although neither recall performance nor cycling consistency differed between sexes.

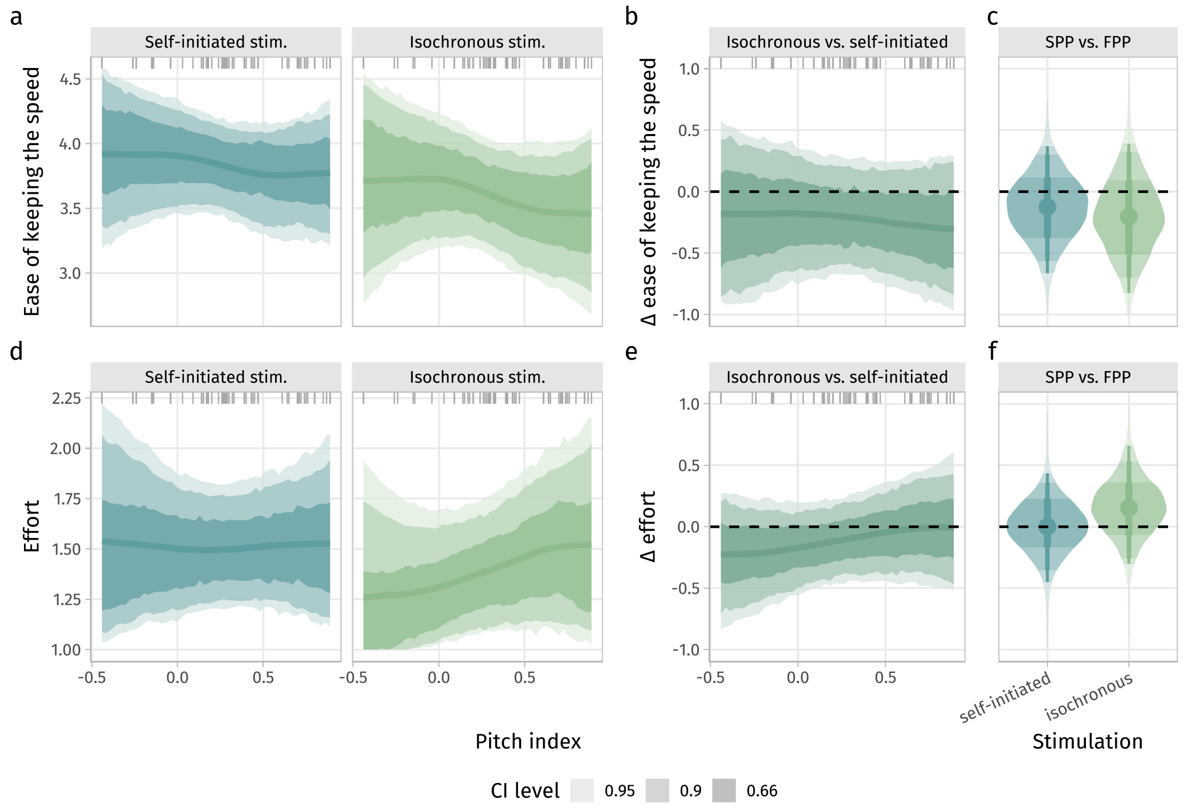

Suppl. Figure 8. Inverse task difficulty (top row) and perceived physical effort (lower row) as a function of pitch perception preference (x-axis) and stimulation condition (facets). Subjects with more dominant spectral pitch perception (SPPs) perceived the isochronous (AMS) condition as slightly more difficult (a) and effortful (d) than subjects with more rate-related pitch perception (FPPs), albeit this was only a rather uncertain trend (binned contrasts between the higher and lower half of pitch indices in c) and f), respectively). All subjects found the isochronous (AMS) condition slightly more difficult than the self-initiated (b), while only FPPs found it less effortful (e).

Musical experience (years of musical training) initially increased perceived difficulty of the AMS task over the first 10 years, possibly reflecting increasing self-awareness of the sensorimotor alignment with musical training (suppl. Fig. 9a, right facet) and decreased again with more musical experience, possibly reflecting advantages of musical training for the AMS task (suppl. Fig. 9A and c). Interestingly, this pattern was mirrored in perceived effort, as longer musical experience went along with decreased perceived physical effort of the AMS task over the first 10 years of musical training, possibly an attentional trade-off between a higher

awareness of the cognitive challenge of active audio-motor synchronization and the perception of the physical effort of cycling (suppl. Fig. 9d-f). However, across subjects, perceived task difficulty and effort were slightly *positively* correlated (ρ of the correlation between the multivariate group-level (i.e. individual) intercepts of ease of keeping the cycling speed and effort ≈ -0.22 [-0.63, 0.31], pd ≈ 0.79, BF ≈ 1.3). Individuals rating the task as easier performed also slightly better in the vocabulary tests (ρ of the correlation between the group-level intercepts ≈ 0.17 [-0.32, 0.60], pd ≈ 0.76, BF ≈ 1.2), while no correlation was observed between effort rating and recall performance (pd ≈ 0.6).

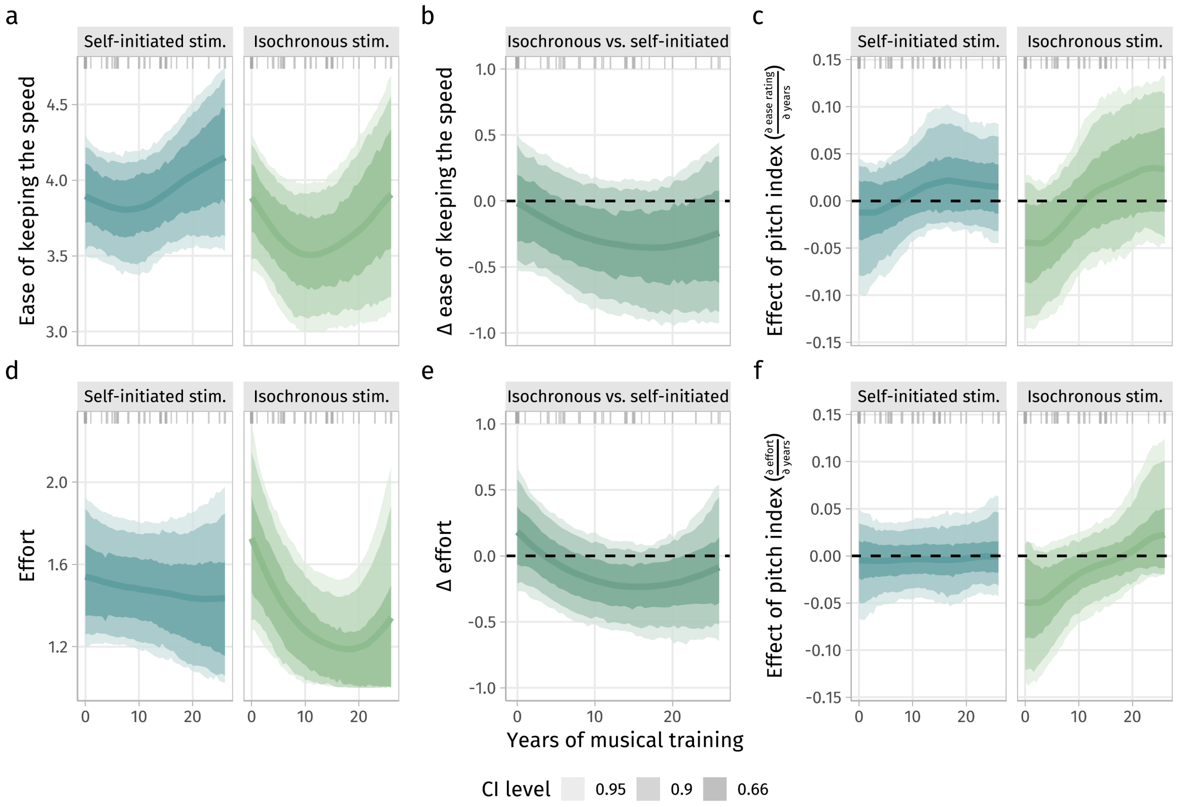

Supp. Figure 9. Inverse task difficulty (top row) and perceived physical effort (lower row) as a function of musical experience (x-axis) and stimulation condition (facets). Musical experience (years of musical training) initially increased perceived difficulty of the AMS task over the first 10 years and decreased again with more musical experience (U-shaped pattern in (a) and a linear zero-crossing pattern in the partial derivative in (c)). Musical experience went along with decreased perceived physical effort of the AMS task over the first 10 years ((d) and (f)).

Inter-subject variability and subject specific "traits"

While recall performance was rather consistent within subjects over the four Polish vocabulary tests (intra-class correlation, ICC ≈ 0.56 [0.27, 0.72] (1 indicating all 4 scores were identical)), and between the pre-screening pseudo vocabulary test and the Polish vocabulary tests (ρ of the

multivariate random-intercept correlation ≈ 0.57 [0.28, 0.79], pd ≈ 1.00), recall performance varied considerably between subjects in a way only partly explained by the observed and modeled factors, as reflected in a SD of the Gaussian distribution over group-level intercepts of 0.65 [0.05, 0.83] on the log odds scale (corresponding to ~ +/-66% probability; suppl. Fig. 10).

Also the effect of condition varied non-systematically over subjects (SD ≈ 0.56 [0.36, 0.78]). Interestingly, the individual condition effect was slightly positively correlated with a subject's mean performance, such that a higher average performance (individual intercept) co-occurred with a larger positive difference between the isochronous and the self-initiated stimulation, an effect not captured by a population level effect of either of the behavioral metrics or any other covariate (correlation between the group-level intercept and the condition effect in the pitch index model $\rho \approx 0.22$ [-0.13, 0.56], pd ≈ 0.88; in the speed model: $\rho \approx 0.19$ [-0.20, 0.55], pd ≈ 0.82; in the CV model: $\rho \approx 0.24$ [-0.15, 0.63], pd ≈ 0.86; in the IBD model: $\rho \approx 0.22$ [-0.16, 0.56], pd ≈ 0.87).

The data lent little support for between-subject variability of the effects of testing day (SD ≈ 0.06 [0.00, 0.18]) and sleep (SD ≈ 0.08 [0.00, 0.25]) on memory recall and similarly for the within-subject variability in the decomposed motor metrics in the respective models.

Motor performance also varied strongly between subjects in ways not explainable by the observed covariates (speed SD ≈ 0.87 [0.52, 1.23] Hz, CV SD ≈ 0.28 [1.7, 0.41] (log-scale), IBD (an observation-level random effect) SD ≈ 0.72 [0.47, 0.93] (log-scale)). Individual deviation from mean pedaling speed was strongly positively correlated with deviation from mean IBD ($\rho \approx 0.41$ [0.05, 0. 47, pd ≈ 0.98), reflecting the fact that IBD was primarily driven by pedaling faster than the stimulation rate (as evident in the global intercept of speed being > 1 Hz); individual variability in pedaling speed was slightly negatively correlated with CV variability. Individual variability between IBD and CV were also strongly correlated ($\rho \approx 0.38$ [0.02, 0. 70], pd ≈ 0.97), likely due to the mathematical relatedness between the two metrics.

More interestingly, individual differences in average memory performance were slightly negatively correlated with individual deviations in CV ($\rho \approx$ *-0.24 [-0.59, 0.11], pd ≈ 0.90*) and IBD ($\rho \approx$ *-0.12 [-0.42, 0.21], pd ≈ 0.76*), but not with speed variability ($\rho \approx 0.02$*, pd ≈ 0.54*), corroborating the findings in the separate regression models with these metrics as population level predictors. Individual CV variability was slightly positively correlated with deviations from the mean stimulation effect on memory recall ($\rho \approx 0.30$ *[-0.12, 0.68], pd ≈ 0.92*), a relation

not detected when fitting a population-level effect of log CV in the separate regression model, which indicates that individuals with a high (intrinsic?) motor variability benefited more from the regular motor-independent vocabulary presentation than subjects with lower individual CV. However, there may be alternative explanations for this, e.g. a mediating effect of musical experience (which was associated with both a higher average CV and better memory performance) together with the described correlation between individual mean performance and individual condition effects.

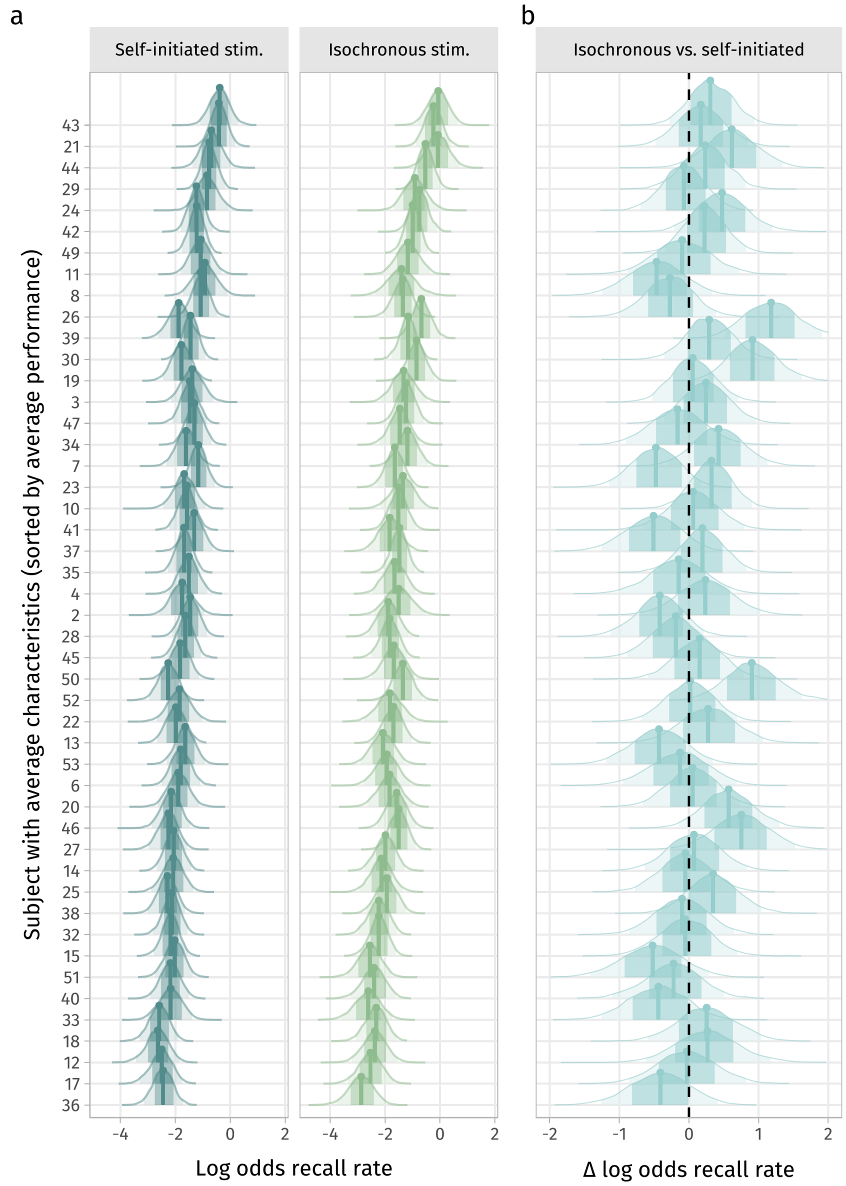

Suppl. Figure 10. Variation in absolute (a) and relative (isochronous vs. self-initiated stimulation; b) log odds recall rate (i.e. on the link scale) over subjects, which is not explained by any other systematic (population level) characteristic. Sampling variability is also not included (as in all other Figures). The more positive the log-odds value, the higher the probability of successful recall. Subjects are arranged vertically by decreasing average memory performance.

Extended discussion

Average cycling speed above target. A surprising result was the consistently (on average 7%) higher pedaling rate than the auditory stimulus pace of 60 rpm—especially among musically less experienced participants. Even more puzzling, participants pedaled faster in the isochronous than in the self-initiated condition, despite the fixed rhythm presumably exerting stronger temporal constraints. This effect warrants further investigation. Possible explanations may include the influence of an intrinsic tempo attractor above 1 Hz, or anticipatory synchronization mechanisms [14,15], though it is unclear why such effects would persist over the full 20-minute session. Notably, time of day did not systematically affect this pattern.

Effects of sleep. We observed that subjects sleeping longer on average cycled closer to the target speed of 1 Hz and displayed lower speed variability (CV) and higher synchronization (IBD) (but slightly worse memory performance; see suppl. Fig. 7). Considering that both absolute CV and speed as well as effects of stimulation condition were modulated by musical experience (with subjects with more musical experience being more strongly affected by the different stimulation regimes), it seems plausible that sleep may have a positive functional influence on sensorimotor synchronization. However, within-subject sleep variation between the two sessions did not additionally explain variation in motor performance, likely because the low number of observations per subject (only 2) precluded the reliable estimation of within-subject associations.

References

1. Bürkner, P. C. & Vuorre, M. Ordinal Regression Models in Psychology: A Tutorial. *Adv Methods Pract Psychol Sci* **2**, 77–101 (2019).

2. Vehtari, A., Gelman, A. & Gabry, J. Practical Bayesian model evaluation using leave-one-out cross-validation and WAIC. *Stat Comput* **27**, 1413–1432 (2016).

3. Gelman, A., Goodrich, B., Gabry, J. & Vehtari, A. R-squared for Bayesian Regression Models. *Am Stat* **73**, 307–309 (2019).

4. Gelman, A. & Rohilla Shalizi, C. Philosophy and the practice of Bayesian statistics. (2012) doi:10.1111/j.2044-8317.2011.02037.x.

5. Shmueli, G. To Explain or to Predict? *https://doi.org/10.1214/10-STS330* **25**, 289–310 (2010).

6. Cinelli, C. & Hazlett, C. Making Sense of Sensitivity: Extending Omitted Variable Bias. *J R Stat Soc Series B Stat Methodol* **82**, 39–67 (2020).

7. Schneider, P., Sluming, V., Roberts, N., Bleeck, S. & Rupp, A. Structural, functional, and perceptual differences in Heschl's gyrus and musical instrument preference. *Ann N Y Acad Sci* **1060**, (2005).

8. Schneider, P. *et al.* Structural and functional asymmetry of lateral Heschl's gyrus reflects pitch perception preference. *Nat Neurosci* **8**, 1241–1247 (2005).

9. Ladd, D. R. *et al.* Patterns of individual differences in the perception of missing-fundamental tones. *J Exp Psychol Hum Percept Perform* **39**, (2013).

10. Seither-Preisler, A. *et al.* Tone sequences with conflicting fundamental pitch and timbre changes are heard differently by musicians and nonmusicians. *J Exp Psychol Hum Percept Perform* **33**, 743–751 (2007).

11. Schneider, P. *et al.* Neuroanatomical Disposition, Natural Development, and Training-Induced Plasticity of the Human Auditory System from Childhood to Adulthood: A 12-Year Study in Musicians and Nonmusicians. *J Neurosci* **43**, (2023).

12. Bücher, S., Bernhofs, V., Thieme, A., Christiner, M. & Schneider, P. Chronology of auditory processing and related co-activation in the orbitofrontal cortex depends on musical expertise. *Front Neurosci* **16**, 1041397 (2023).

13. Gruhn, W., Hofmann, E. & Schneider, P. Grundtonhörer? Obertonhörer? Hörtypen und ihre Instrumente.

14. Roman, I. R., Washburn, A., Large, E. W., Chafe, C. & Fujioka, T. Delayed feedback embedded in perception-action coordination cycles results in anticipation behavior during synchronized rhythmic action: A dynamical systems approach. *PLoS Comput Biol* **15**, (2019).

15. Repp, B. H. & Su, Y. H. Sensorimotor synchronization: a review of recent research (2006-2012). *Psychon Bull Rev* **20**, 403–452 (2013).
